# Nanoparticle delivery systems for gene therapy of degenerative musculoskeletal diseases

**DOI:** 10.1016/j.jot.2026.101241

**Published:** 2026-10-25

**Authors:** Xiaofei Chen, Jiale Sun, Wenqing Xie, Djandan Tadum Arthur Vithran, Qidong Zhang, Jessica M. Rosenholm, Yusheng Li, Hongbo Zhang, Cheng Huang

**Affiliations:** a Deparment of Orthopedics, Xiangya Hospital, Central South University, Changsha, Hunan, 410008, China; b Xiangya School of Medicine, Central South University, Changsha, Hunan, 410083, China; c Health Science Center, Peking University, Beijing, 100191, China; d National Clinical Research Center for Geriatric Disorders, Xiangya Hospital, Central South University, Changsha, Hunan, 410008, China; e Department of Orthopedics, China-Japan Friendship Hospital, Beijing, 100029, China; f Pharmaceutical Sciences Laboratory, Faculty of Science and Engineering, Åbo Akademi University, Turku, 20500, Finland; g Turku Bioscience Centre, University of Turku and Åbo Akademi University, Turku, 20014, Finland

**Keywords:** Ageing, Degenerative musculoskeletal diseases, Delivery systems, Gene therapy, Nanoparticles, Osteoarthritis

## Abstract

Degenerative musculoskeletal diseases (DMDs) are a group of chronic conditions characterized by the progressive deterioration of tissue structure and function, primarily affecting the articular cartilage, bones, tendons, and ligaments. With global aging populations, the rising prevalence of these disorders presents a growing public health challenge. Current treatments for DMDs are limited by substantial drug side effects and restricted therapeutic efficacy. Gene therapy has emerged as a promising alternative for addressing these conditions. Nanoparticles are essential for overcoming intracellular and extracellular barriers in nucleic acid delivery. Recent technological advances have led to novel nanomaterials and strategies, which have enabled safer and more efficient gene therapy. This review comprehensively analyzes the role of nanoparticle delivery systems in enabling effective gene-based interventions for major DMDs. First, we analyze the main gene therapies for various DMDs. Next, we systematically summarize recent advances in nanocarrier platforms and delivery strategies targeting osteoporosis, osteoarthritis, intervertebral disc degeneration, and tendon diseases. Finally, we discuss key challenges and future directions, offering a roadmap to advance nanoparticle delivery therapeutics toward clinical impact. This synthesis uniquely bridges nanoparticle delivery design with disease-specific pathophysiology, providing strategic guidance for overcoming translational barriers through emerging technologies including AI-driven carrier optimization and organ-on-chip platforms.

Across these diseases, no carrier is universally optimal: systemic bone delivery prioritizes circulation stability and cell-selective targeting, whereas local delivery to cartilage, intervertebral discs, and tendons requires a balance among tissue retention, matrix penetration, cellular uptake, and controlled release. Current evidence remains predominantly preclinical and frequently demonstrates tissue accumulation without fully establishing intact-cargo delivery, functional target engagement, long-term safety, or manufacturability. This review therefore links nanoparticle design to disease-specific barriers and identifies the evidence required for clinical translation.

The Translational Potential of this Article.

This review focuses on the clinical translational potential of nanoparticle-based delivery systems for gene therapy in degenerative musculoskeletal diseases (DMDs). Addressing the unique physiological and pathological barriers of specific musculoskeletal tissues (such as bone, cartilage, intervertebral discs, and tendons), this article systematically summarizes targeting and sustained-release strategies designed to enhance delivery efficiency and overcome tissue-specific obstacles. Furthermore, this study highlights future perspectives, including artificial intelligence (AI)-driven carrier optimization, organ-on-a-chip models, and multimodal combination therapies integrating biomaterials and cells. By addressing the limitations of conventional therapies that merely alleviate symptoms without reversing tissue degeneration, this review provides a robust theoretical foundation and a clear translational roadmap for the development of precise, safe, and long-lasting gene therapy regimens for DMDs.

## Graphical abstract

Overview of nanoparticle delivery systems applied to gene therapy for degenerative musculoskeletal diseases.

**Figure:**
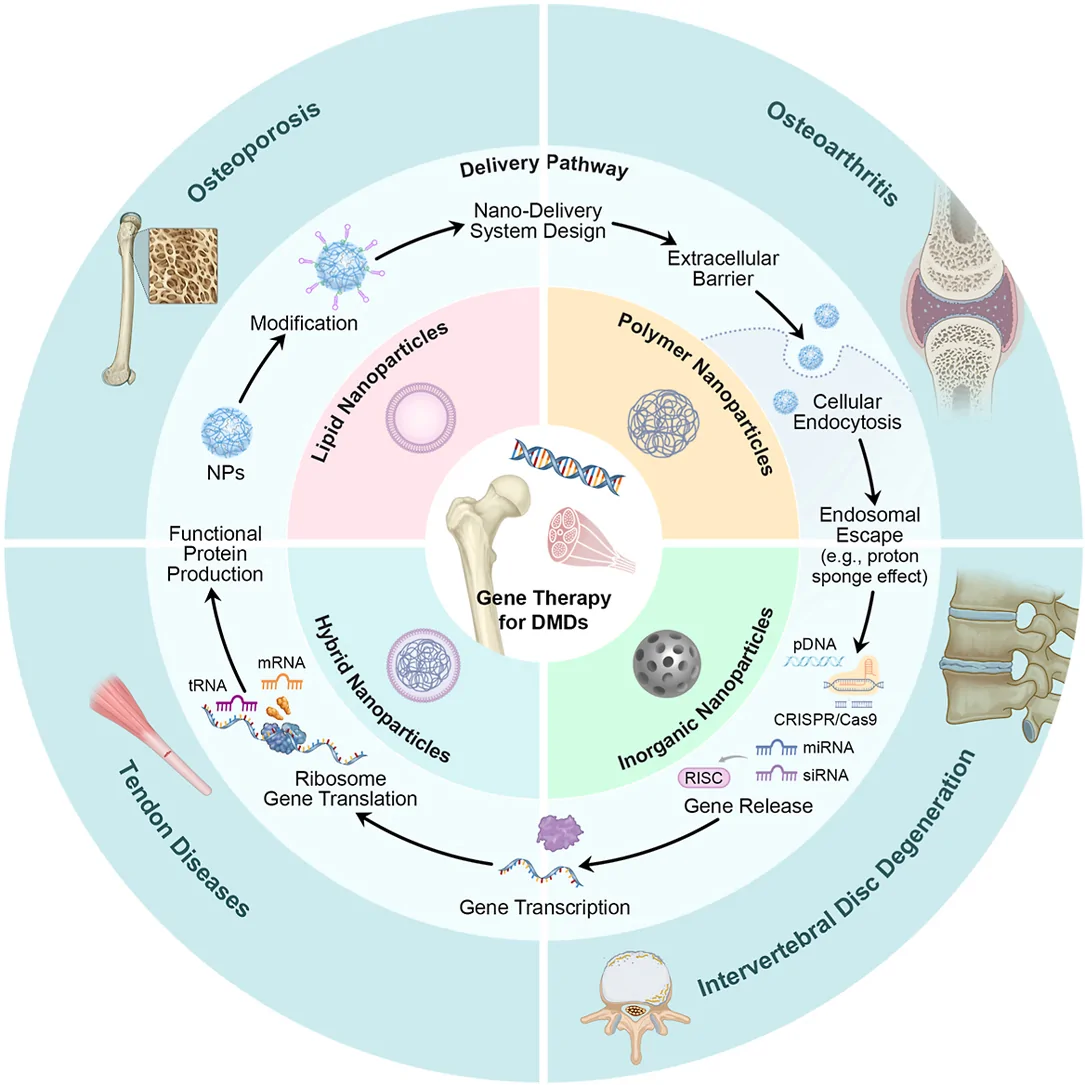

## 1 Introduction

Degenerative musculoskeletal diseases (DMDs) include various age-related disorders of the musculoskeletal system, such as osteoporosis (OP), osteoarthritis (OA), intervertebral disc degeneration (IDD), and tendon diseases. These diseases cause patients to experience pain, impaired mobility, and significant decline in their health, social functioning, and quality of life. In addition, the treatment of diseases leads to increased financial burden on families [1]. According to the World Population Ageing Priorities 2020 report, by 2020, 727 million people worldwide were aged 65 years and above. The trend of population aging is accompanied by a significant increase in the prevalence of musculoskeletal diseases, which is expected to grow further in the future [2]. This trend imposes growing socio-economic and clinical burden on global healthcare systems. Pharmacological treatments for degenerative musculoskeletal diseases mainly focus on pain relief or symptomatic management and are often accompanied by significant systemic side effects. More importantly, they fail to effectively reverse structural degeneration or halt disease progression at the molecular and pathological levels [3]. Surgical interventions, such as intervertebral disc surgery and joint replacement, are primarily applied for structural reconstruction or symptom alleviation; however, they are invasive, associated with long recovery periods, and do not fundamentally restore cellular function or correct underlying molecular abnormalities [4]. Cell therapy has shown great potential in the repair and regeneration of damaged cartilage and osteochondral integration zones; however, it may face challenges such as low cell viability, donor cell variability, and immune rejection [3].

These persistent bottlenecks necessitate the development of a more fundamental therapeutic approach: gene therapy. Gene therapy is an emerging technology that involves the use of nucleic acids (DNA or RNA) to treat, cure, and prevent human diseases. Gene therapy has multiple advantages, allowing for more precise and long-lasting disease treatment, as well as the potential for personalized treatments. With a better understanding of the etiology of DMDs and the development of gene delivery technologies, gene therapy may become a potential treatment option. However, the clinical translation of gene therapy for DMDs is profoundly constrained by delivery challenges inherent to musculoskeletal tissues. Dense extracellular matrices, poor vascularization, and biologically hostile environments impede effective transport, cellular uptake, and sustained expression of nucleic acid cargos. Moreover, naked nucleic acids are susceptible to rapid enzymatic degradation and lack intrinsic tissue targeting, making efficient in vivo delivery extremely difficult [50]. To overcome these barriers, nanoparticle-based delivery systems have emerged as enabling platforms [51]. To appreciate the rationale for nanoparticle-based approaches, it is essential to contrast them with viral delivery systems. Viral vectors (lentiviruses, adenoviruses, adeno-associated viruses) have historically dominated gene delivery due to their high transduction efficiency. However, they face substantial challenges: limited cargo capacity (<8 kb for AAV), risk of insertional mutagenesis, immunogenicity concerns, and complex manufacturing processes [52]. In contrast, non-viral nanoparticle carriers offer distinct advantages: (1) higher payload capacity accommodating diverse nucleic acid types (siRNA, miRNA, mRNA, pDNA, CRISPR components); (2) lower immunogenicity and improved safety profile; (3) flexible surface modification for targeted delivery; (4) easier manufacturing and scalability; and (5) greater design versatility to match tissue-specific requirements. Despite these advantages, achieving comparable transfection efficiency remains the primary challenge driving current nanoparticle research [53]. Prior reviews remain limited to specific diseases, such as functionalized nanoparticles for OP [54] or the "aging-metabolism-inflammation" axis in OA [55]. Although gene delivery in arthritis and bioengineered nanozymes are well-reviewed [56], [57], they overlook the broader spectrum of degenerative musculoskeletal diseases (DMDs). Crucially, a synthesis aligning nanoparticle design with the distinct biological barriers across OP, OA, IDD, and tendon diseases is still lacking.

Therefore, this review focuses not simply on summarizing nanomaterials or gene therapy in isolation, but on comprehensively analyzing the roles of nanoparticle delivery systems in enabling effective gene-based interventions in major degenerative musculoskeletal diseases (Graphical abstract). We begin by examining the pathological foundations and gene therapy targets for each disease category. Subsequently, we provide a systematic evaluation of nanocarrier platforms—from lipid-based systems to polymeric nanoparticles, inorganic nanoparticles, and hybrid architectures—analyzing their design principles, targeting mechanisms, and delivery performance in disease-specific contexts. We conclude by identifying critical translational hurdles and proposing actionable strategies to accelerate the clinical development of nanoparticle delivery therapeutics for degenerative musculoskeletal diseases.

## 2 Methodology

### 2.1 Search strategy

For this review, we used the following keywords to search for literature published before June 2026 in the PubMed, Web of Science, Google Scholar, and Scopus databases, in order to secure comprehensive coverage of major research studies.

A combination of keywords was used for the search, including "Nanoparticle," "Nanomaterial," "Gene Therapy," "Degenerative Musculoskeletal Diseases," "Osteoporosis," "Osteoarthritis," "Intervertebral Disc Degeneration," "Tendon Diseases," "siRNA," "miRNA," "CRISPR/Cas9," "Non-viral vectors," "Delivery System," "Targeted Delivery," and "Translational Barriers." To ensure no significant research in this field is overlooked, the aforementioned keywords were frequently used either in paired combinations or individually when conducting searches. Detailed descriptions of the specific search strategies are omitted herein.

The literature search focused on the most up-to-date research results in this field over the last 5 years, while additionally integrating classic earlier researches that hold great significance for the discipline. Only English-language publications were included to guarantee both readability and global academic relevance.

### 2.2 Inclusion and exclusion criteria

The inclusion criteria specifically cover original research (including both in vitro experimental and in vivo animal models) as well as high-quality review articles, along with studies that focus on the structural design, targeting mechanisms, and cellular interactions underlying the role of nanoparticle-mediated gene therapy in degenerative musculoskeletal diseases. The exclusion criteria include studies unrelated to nanocarriers, viral-vector gene therapies, non-musculoskeletal diseases, and publications that lack complete data or an adequate experimental design.

### 2.3 Literature screening process

First, for the initial screening, researchers screened relevant literature based on titles and abstracts, excluding articles that were clearly irrelevant. Secondly, for the full-text review, articles that potentially met the inclusion criteria underwent further evaluation to verify their compliance with the criteria. Finally, for dispute resolution, in cases of disagreements arising during the screening process, three researchers engaged in joint discussion to ensure the objectivity of the literature selection.

## 3 Nanotherapeutic targeting strategies for DMDs

### 3.1 Degenerative musculoskeletal diseases and their core pathological characteristics

Degenerative musculoskeletal diseases (DMDs) are widely regarded as typical age-related conditions, with their onset and progression closely linked to aging. Aging is a complex degenerative process involving multiple systemic functions and is a significant risk factor for degenerative diseases. Studies have indicated that the accumulation of senescent cells in aging tissues is a key factor in triggering degenerative diseases [58], [59], [60], [61]. These senescent cells are characterized by cellular genetic mutations, telomere shortening, protein aggregation and misfolding, and excessive production of reactive oxygen species (ROS) (Fig. 1) [62]. The interaction between senescent cells and Senescence-Associated Secretory Phenotype‌ (SASPs) accelerates the aging process, ultimately leading to cellular dysfunction, matrix remodeling, chronic inflammation, and other pathophysiological changes, thereby promoting degenerative processes. With an aging population, the prevalence of musculoskeletal disorders is expected to continue to increase in the future, making it a major public health challenge in an aging society [2], [63].

**Fig. 1:**
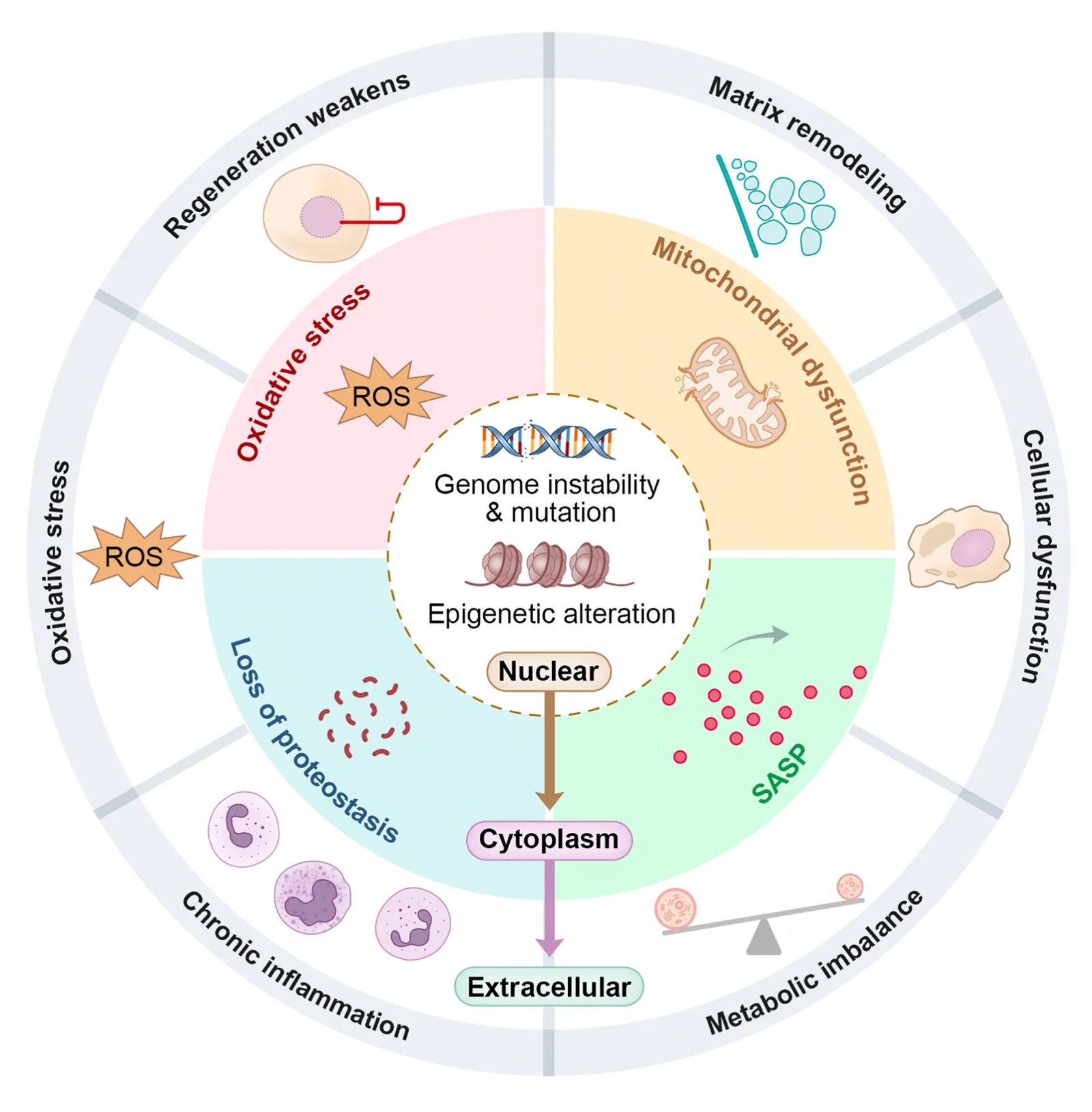
Mechanisms by which aging promotes degenerative diseases. The occurrence of degenerative diseases is related to physiological aging (age increase) and pathological aging (pathological damage, such as poor living habits, strain, trauma, and environmental factors). SNCs (characterized by increased ROS, mitochondrial dysfunction, genome instability and mutation, epigenetic alteration, loss of proteostasis, etc.) secrete SASP (including pro-inflammatory cytokines, chemokines, growth factors, matrix metalloproteinases, etc.). The interaction between SNCs and SASP causes cellular dysfunction, matrix remodeling, chronic inflammation, weakened regeneration, metabolic imbalance, and oxidative stress, ultimately leading to degenerative diseases.

### 3.2 Strategy selection: Matching nanoparticle delivery to disease pathophysiology

With the continuous development of science and technology, gene therapy has gradually shown broad application prospects and unique advantages in the treatment of musculoskeletal diseases. Currently, gene replacement, gene addition, gene editing, gene suppression, and inactivation are the four strategies commonly used in gene therapy [64].

#### 3.2.1 Gene addition strategies and delivery considerations

One of the main strategies used in nanoparticle delivery system-based gene therapy for DMDs is the gene addition approach, in which therapeutic genes are introduced into target cells and then expressed to reduce damaging factors such as inflammation or to modulate anabolic and catabolic pathways that are essential for tissue repair. The therapeutic genes selected for OP, OA, and other diseases involving bone and cartilage tissues are usually osteogenic and chondrogenic-related growth factors and transcription factors, mainly including bone morphogenetic proteins 2/4/7 (BMP2/4/7), osteoprotegerin (OPG), vascular endothelial growth factor (VEGF), and fibroblast growth factor 8 (FGF18) and so on [65], [66], [67], [68], [69], [70]. In addition, studies have also been conducted to modulate the homeostasis of the immune system and suppress inflammation in the musculoskeletal system by delivering anti-inflammatory response-related genes, such as IL-1 Ra and IL-10 [71], [72]. Vectors that express genes can be directly injected into the body, which is called in vivo gene therapy. It is also possible to transplant therapeutic genes into mesenchymal stem cells (MSCs) or adipose-derived stem cells (ADSCs) and then implant these modified cells into the body, which is called in vitro gene therapy [73].

#### 3.2.2 Gene suppression and inactivation strategies: advantages of RNAi-based approaches

In recent years, gene therapy research for DMDs using nanoparticle delivery systems has gradually focused on gene suppression and inactivation strategies, in which RNAi technology, including siRNAs and miRNAs, plays an important role. Among the currently available nucleic acid therapeutics, siRNA is the most extensively investigated delivery cargo in DMD-related studies, largely owing to the high compatibility between its site of action and non-viral delivery mechanisms.

Compared with siRNA, other nucleic acid modalities impose more stringent requirements on delivery systems. Although mRNA also functions in the cytoplasm, its larger molecular size and lower structural stability necessitate enhanced carrier-mediated protection and highly efficient endosomal escape for effective intracellular delivery [74]. Meanwhile, plasmid DNA (pDNA) must further traverse the nuclear envelope to enable transcription, and this nuclear transport process is widely regarded as one of the most challenging biological barriers for non-viral gene delivery systems, particularly in musculoskeletal tissues characterized by low cellular proliferative activity and dense extracellular matrices [75].

By comparison, siRNA exerts its gene-silencing effect directly in the cytoplasm through association with the RNA-induced silencing complex (RISC), thereby bypassing the requirement for nuclear entry, which represents the most formidable obstacle in non-viral gene delivery. Consequently, as long as a delivery system can achieve efficient cellular uptake and successful endosomal escape to release siRNA into the cytoplasm, its therapeutic activity can be readily initiated. This mechanistic feature confines the primary design requirements of siRNA carriers to cellular internalization and endosomal escape rather than complex nuclear-targeting strategies, thereby substantially enhancing the feasibility of siRNA-based therapies in hard-to-transfect tissues, such as those involved in DMDs [76].

Negative regulators of osteogenic pathways (e.g., Sema4d, noggin, and Wwp1) and positive regulators that mediate osteoclast bone resorption (e.g., RANK pathway) have emerged as potential targets of RNAi technology for the treatment of OP [77], [78], [79], [80]. siRNA-mediated knockdown of these genes enables precise modulation of bone remodeling processes without introducing exogenous gene expression, thereby reducing the risk of long-term safety concerns.

In addition, chronic inflammation, oxidative stress, fibrosis, and apoptosis are often pathological changes that accompany injury to the bone, cartilage, intervertebral discs, and tendons, which promote matrix degradation and cell death while inhibiting cellular anabolism, thereby inhibiting tissue regeneration [81]. Numerous studies have demonstrated that RNAi can target key pathways involved in these pathological alterations, thereby restoring the healthy microenvironment required for tissue repair and demonstrating promising results in the treatment of DMDs.

#### 3.2.3 Emerging strategies: CRISPR/Cas9-based therapies

The development of CRISPR/Cas9 technology has opened new avenues for simple and efficient gene editing. However, CRISPR/Cas9 technology also means that the contents become more numerous, thereby increasing the complexity of delivery. As a result, few studies have been conducted to deliver CRISPR/Cas9 systems for the treatment of DMDs. Recently, Lin et al. developed an acid-responsive and neutralizing system with high in vivo gene editing capacity by loading sodium bicarbonate (NaHCO3) and RANKL-CRISPR/Cas9 (RC) plasmid in metal-organic nanoparticles for bone protection by promoting osteogenesis and inhibiting osteolysis, illustrating the potential of CRISPR/Cas9 technology in DMDs [27].

## 4 Nanoparticle platforms for musculoskeletal gene delivery

### 4.1 Extracellular and intracellular delivery barriers

Although promising, several challenges need to be considered to further advance the role of gene therapy in DMDs: ① limited tissue specificity; ② biological barriers limiting gene and vector entry, which can be categorized as extracellular delivery and intracellular transport; ③ safety issues, such as immune response and long-term effects; and ④ short-term effects and off-target effects of gene therapy [51], [82].

First, before reaching target cells, delivery systems must overcome a series of extracellular and tissue-level barriers, including complex anatomical structures, dilution and clearance by body fluids, nuclease-mediated degradation, insufficient particle stability, and nonspecific protein adsorption. These factors can markedly reduce the effective nucleic acid concentration at the target site, making precise delivery particularly challenging in musculoskeletal tissues characterized by limited vascularization, sparse cellular distribution, and dense extracellular matrices [76].

After successfully reaching the target tissue, intracellular delivery barriers become the critical determinants of therapeutic efficacy. Non-viral nanocarriers are typically internalized by cells via endocytosis and subsequently sequestered within endosomes. During endosomal maturation, progressive acidification and enrichment of lysosomal enzymes occur; if the delivery system fails to achieve effective escape at this stage, the encapsulated nucleic acids are highly susceptible to degradation, resulting in loss of biological activity [75], [83].

Different types of nanocarriers exhibit pronounced differences in their ability to overcome this barrier. For example, certain cationic polymers can induce increased osmotic pressure within endosomes through the “proton sponge effect”, leading to membrane disruption, whereas lipid-based carriers tend to promote cytosolic release of nucleic acids via membrane fusion or membrane perturbation mechanisms. In contrast, material systems lacking effective endosomal escape capability may display severely compromised overall transfection efficiency, even if they demonstrate favorable cellular uptake [83].

Moreover, As discussed in Section 3.2.2 the nature of delivery barriers varies substantially among different types of nucleic acids [75], [76].

### 4.2 Nanocarrier classes and design trade-offs

These hurdles restrict the clinical translation of in the application of gene therapy to DMDs. Therefore, next-generation delivery materials must be developed to overcome these challenges. Viral gene delivery methods, including lentiviruses, other retroviruses, adenoviruses, and adeno-associated viruses, have largely dominated the field of gene delivery because of their high efficiency of cell transduction. However, issues such as manufacturability, immune response, toxicity, and limited cargo capacity remain [50], [84]. Nanoparticles, as non-viral vectors, address these challenges through their unique physicochemical properties and are gradually moving from the laboratory to the clinic as a disruptive technology in the field of gene therapy, owing to their low toxicity, low immune response, high cargo capacity, flexibility, and ease of production and storage, which is expected to lead to a breakthrough in gene therapy for DMDs in the future [85].

In addition, nanoparticle carriers have additional design space to personalize and adjust their structure and characteristics according to the type of nucleic acid delivered, target tissue, and type of disease, which is mainly aimed at the following aspects: ① Cargo capacity: Flexible nucleic acid cargo capacity can be obtained by electrostatic binding between large anionic nucleic acids and cationic biomaterials. ② Particle size: The nanoparticle size must be adjusted to the optimal diffusion range according to the matrix gap pore size of each tissue. Especially for dense bone matter, cartilage, and tendon tissue, smaller particles are more likely to penetrate the matrix to reach target cells. ③Targeting capacity: Ligand modifications targeting specific receptors in skeletal muscle tissue (e.g., peptides, antibodies, and polysaccharides) can significantly improve the tissue/cell targeting of nanoparticles and reduce off-target effects. ④Biodegradability of the carrier: Musculoskeletal tissues are mostly slow-renewal tissues (such as bone and cartilage renewal cycles of months to years), and non-degradable nanoparticles are prone to accumulate in tissues and cause chronic toxicity. The carrier can be degraded through enzymatic cleavage of various chemical bonds within the biomaterial structure (e.g., ester bonds and disulfide linkages), thereby facilitating cargo release, improving therapeutic efficacy, and enhancing biosafety. ⑤Cargo release timing and mode: Vectors can be designed to biodegrade rapidly upon reaching the reducing environment of the cytoplasm, releasing biological cargoes within the cell in an environmentally triggered manner, which can reduce the loss of therapeutic genes and achieve a sustained and tunable release of genes.

The commonly used nanoparticle delivery vehicles can be divided into the following categories: lipid nanoparticles, polymer nanoparticles, inorganic nanoparticles, and hybrid nanoparticles(Table 1) [86].

**Table 1:** Gene delivery systems for musculoskeletal degenerative diseases.

Nanomaterials	Advantages	Limitation	Delivery System	Diseases	Ref.
Lipids	Low immunogenicity; Good biocompatibility; Good biodegradability	Low stability; Short half-time in the bloodstream; Potential toxicity; Poor targeting ability	Cationic liposomes based on DOTAP and (AspSerSer)6	OP	[5]
			LNP delivery system using the novel ionizable lipids	OP	[6]
			LNPs based on Dlin-KC2-DMA	OA	[7]
			Rationally optimized SM-102-based LNPs	OA	[8]
			Cationic liposomes	IDD	[9]
PLGA	Good biocompatibility; Controllable andmodifiabledegradationproperties; Small molecular weight; Ability to be modifiedwith active targetingligands	Low encapsulation efficiency and loading rates	Poly (lactic-co-glycolic acid) (PLGA)-based Nanoparticles	OA	[10], [11]
			Polyvinylamine (PVAm) modified by poly lactic-co-glycolic acid (PLGA) and cartilage-targeting peptide (CAP)	OA	[12]
			A nanoparticle loaded with a specific aptamer (PLGA-PEI-PEG-RD4)	IDD	[13]
			Polylactic-co-glycolic acid nanospheres	Tendon diseases	[14]
			PEI-modified polylactic-co-glycolic acid (PLGA) nanoparticles	Tendon diseases	[15]
			a novel platform based on the biocompatible polylactic-co-glycolic acid (PLGA) nanoparticle and hyaluronic acid (HA) hydrogel	Tendon diseases	[16]
Chitosan	Good biocompatibility; Low toxicity; High positive charge Ability to be modified with active targeting ligands	Poor water solubility; Poor targeting ability; A lack of endosomal escape mechanisms; Low gene transfection efficiency	Chitosan/siCkip-1 biofunctionalized titanium	OP	[17]
			Chitosan–DNA nanoparticles	OA	[18]
			Nano-microspheres containing chitosan, hyaluronic acid, and chondroitin sulfate	OA	[19]
Polyethyleneimine (PEI)	Endosomal escape capacity; Strong drug-carrying capacity; Ability to be modified with active targeting ligands	High cytotoxicity	Dexamethasone-conjugated polyethyleneimine (DEXPEI) nanoparticles	OA	[20]
Inorganic Nanoparticles	High surface area-to-volume ratio; Strong drug-carrying capacity; Ability to be modified with active targeting ligands; Modification allows for traceability and environmental responsiveness	Potential toxicity; Low gene transfection efficiency	MIL-101-NH2 nanoparticles	OA	[21]
			Copper sulfide (CuS) nanoparticles	OA	[22]
			Copper sulfide@phosphatidylcholine nanoparticles	OA	[23]
			Carboxyl-modified MCM-41 mesoporous silica nanoparticle	Tendon diseases	[24]
Lipid-polymer hybrid nanoparticles	Ability to combine biocompatibility with high drug-carrying capacity		Lipid-polymer nanoparticles based on PLGA and DOTAP and functionalized with aptamers	OP	[25]
			TransIT-TKO/siRNA	OP	[26]
Metal-polymer hybrid nanoparticles	Ability to combine environmental responsiveness with high drug-carrying capacity		(Zeolitic imidazolate frameworks-8) ZIF-8 -NaHCO3@Cas9	OP	[27]
			Stem cell membrane-camouflaged (Zeolitic imidazolate frameworks-8) ZIF-8 nanoparticles	IDD	[28]
Inorganic-organic hybrid nanoparticles	Ability to combine strong drug-carrying capacity and high gene transfection efficiency		MSNs modified with an ALN functionalized poly(ethyleneglycol) (PEG) ligand	OP	[29]
			MSNs coated with poly ethylenimine	OP	[30]

### 4.3 Lipid nanoparticles

Lipid nanoparticles (LNPs) are among the most clinically successful nanocarrier platforms and represent the first FDA-approved nanocarriers that specifically enable systemic nucleic acid delivery. They are created by mixing ionizable lipids, amphipathic ionic structured lipids, PEGylated lipids, cholesterol, and nucleic acids [87]. Ionizable lipids, such as DOTMA, DOTAP, or Dimethylaminoethane carbamoyl cholesterol (DC-Chol), enhance nucleic acid encapsulation. They can form complexes with polyanionic nucleic acids through attractive electrostatic interactions, protect nucleic acids from degradation by nucleases, and assist in the entry of nucleic acids into the cell and their release into the cytoplasm [87]. Co-lipids (e.g., DOPE and cholesterol) are used to improve stability and transfection efficiency. They can regulate the size and curvature of the liposome membrane to facilitate the encapsulation of nucleic acids, and can regulate the fluidity of the liposome membrane and charge density to promote the fusion of cellular membranes or endosomal membranes and liposome membranes [88]. Polyethylene glycol (PEG) is commonly used to modify LNPs to increase spatial site resistance, thus preventing liposomes from binding to plasma proteins and prolonging their half-life, allowing the drug to be enriched at the focal site [88].

The clinical success of hepatic LNPs does not directly establish their suitability for musculoskeletal gene delivery because systemic administration remains strongly liver-biased. Recent OA studies have extended LNP application to chondrocyte-targeted SOX5/SOX9 mRNA co-delivery and joint-localized FGF18 mRNA delivery [8], [38]. However, their cartilage penetration, joint retention, and transfection efficiency in large animals remain unclear. Therefore, LNPs for degenerative musculoskeletal diseases require tissue- and route-specific optimization rather than direct extrapolation from vaccines or liver-directed therapeutics.

### 4.4 Polymer nanoparticles

Polymer nanoparticles are attractive alternative platforms for delivering siRNA and miRNA because of their designability and biocompatibility. The representative materials are polylactic acid-hydroxyacetic acid (PLGA), polyethyleneimine (PEI), and chitosan.

#### 4.4.1 PLGA

PLGA is one of the most effective and commonly used biodegradable polymer nanoparticles approved by the FDA in the U.S. [89]. It has excellent biocompatibility and biodegradability and has been used to deliver various drug molecules [90]. Because of their multiple advantages, PLGAs have been widely used as delivery vehicles for gene therapy in the study of various DMDs. First, their small molecular weight makes it easier to penetrate tissues and be taken up by cells [91]. Second, the PLGA matrix can insulate RNA enzymes and guarantee the stability of the delivery system [92]. More importantly, PLGA particles usually have excellent controllable and modifiable degradation characteristics, and the timing of drug release can be modulated by changing the molecular weight, composition of block copolymers, and structure of nanoparticles [90], [93]. However, low encapsulation efficiency and loading rates are their significant limitations.

#### 4.4.2 Chitosan

Chitosan, a natural polysaccharide derived from a wide range of raw materials, with excellent biocompatibility, biodegradability, low immunogenicity, and high positive charge, is the most widely used natural polymer for siRNA delivery. It is often used in gene therapy for OA in the study of DMDs. However, it has some limitations, such as poor solubility at physiological pH, a lack of endosomal escape mechanisms, and low gene transfection efficiency [88]. Several studies have shown that the solubility of chitosan can be improved by hydrophilic modification, and one of the most popular methods is coupling PEG to chitosan. However, PEGylation of chitosan results in lower cellular uptake and reduced transfection efficiency [94]. Therefore, it is crucial to fine-tune the degree of PEGylation to maximize the transfection efficiency of chitosan/nucleic acid nanocomplexes.

#### 4.4.3 Polyethyleneimine

PEI is a synthetic polymer that has long been used for gene transport and has a "proton sponge effect", which enables endosomal escape and facilitates gene release. It has a high positive charge density and molecular weight, and its cationic surface electrostatically binds to nucleic acids, providing an enhanced drug-carrying capacity. In addition, the amino group of PEI facilitates various surface modifications, providing multifunctional or specific targeting sites [95]. However, their high cytotoxicity is a significant drawback. A high-density charge can damage the cell membrane and induce apoptosis, and the higher the molecular weight, the higher the cytotoxicity of PEI. Low molecular weight PEI has lower toxicity but reduced transfection ability [96], [97]. Therefore, researchers have developed many PEI derivatives to reduce cytotoxicity without affecting the transfection ability. For example, cross-linking PEIs with disulfides allows nanoparticles to remain stable until they reach the cell, releasing genes only in the cytoplasm [98]. These significant limitations can be overcome by functionalization with PEG and polycaprolactone (PCL) [99], [100].

### 4.5 Inorganic nanoparticles

Inorganic nanoparticles are promising platforms for nucleic acid delivery in gene therapy. These nanoparticles have high surface area to volume ratios and can load nucleic acid drugs through coupling or electrostatic interactions. They can also achieve lesion-specific aggregation by introducing targeting ligands or functional groups that facilitate binding to nucleic acids or endosomal escape. Their unique physical and optical properties can be used to track inorganic nanoparticles for nucleic acid delivery to cells or tissues [101]. In addition, environmentally responsive nanocarriers can be designed via surface modifications [102]. Gold nanoparticles, magnetic nanoparticles, bioactive glass nanoparticles, hydroxyapatite nanoparticles, and mesoporous silica oxide nanoparticles (MSN) are promising carriers for gene therapy and drug delivery. Among them, MSNs have more applications in gene therapy for DMDs because of their large surface area, high loading capacity, and biocompatibility, and have been proven to be used in gene therapy for OP and tendon diseases. Cationic polymers such as PEI and PEG are commonly used to functionalize MSNs for more cellular uptake [29], [30]. Alternatively, MSNs can be modified with specific amino or carboxyl groups to reduce their toxicity [24], [103].

### 4.6 Hybrid nanoparticles

Hybrid nanoparticles typically refer to nanoparticles made from a combination of different materials, possibly combining organic and inorganic components, which allows for the combination of favorable properties of different materials. Common types include lipid-polymer, metal-polymer, and inorganic-organic hybrid nanoparticles.

#### 4.6.1 Lipid-polymer hybrid nanoparticles

Lipid-polymer hybrid nanoparticles usually consist of a lipid outer layer and a polymer core (e.g., PLGA and chitosan), which combines the biocompatibility of lipids with the high drug-carrying capacity of polymers and further surface modifications such as PEGylation to prolong the in vivo circulation time. It has been shown that lipid-polymer nanoparticles can be effectively used for gene therapy of OP and tendon diseases in DMDs [104], [105].

#### 4.6.2 Metal-polymer hybrid nanoparticles

Metal-polymer hybrid nanoparticles are based on metal nanoparticles (e.g., iron oxide, MSN) as the core and functional polymers (e.g., PEI) wrapped around the outer layer. The metal core confers pH/photo-thermal/magnetic responsiveness, and the polymer shell enhances the gene loading capacity, while the controlled release of contents is achieved by external stimuli (light, magnetic field) or microenvironmental changes (e.g., pH changes) at the lesion site. The pH-responsive metal-organic nanoparticles proved to be capable of delivering not only siRNA but also the CRISPR/Cas9 system successfully, which produced significant therapeutic effects in gene therapy for OA and OP [21], [27].

#### 4.6.3 Inorganic-organic hybrid nanoparticles

Inorganic-organic hybrid nanoparticles are typically represented by a combination of MSNs with lipids or polymers. The mesoporous structure provides a high specific surface area and gene loading, and the organic component improves cellular uptake efficiency. For example, it has been found that PEI-functionalized MSNs improve the biocompatibility of MSNs, while reducing the immunotoxicity of PEIs, and their mediated gene silencing can effectively treat OP [30].

## 5 Osteoporosis: systemic and local bone delivery

### 5.1 Pathogenesis and current status of osteoporosis treatment

Osteoporosis (OP) is a systemic bone disease with various triggers. Owing to the imbalanced state of the biological processes of osteoclasts resorbing old bone and osteoblasts synthesizing and mineralizing new bone matrix, bone resorption in patients is greater than bone formation, which leads to a decrease in bone mineral density and mass, deterioration of the microstructure of the bone tissue, and an increase in bone fragility [106]. Current pharmacological treatments for OP are intended to reduce fragility-fracture risk rather than merely alleviate symptoms. Antiresorptive agents, including bisphosphonates and denosumab, are generally used in patients at high fracture risk, whereas anabolic agents such as teriparatide or romosozumab may be considered for those at very high risk [107], [108]. Nevertheless, long-term adherence, treatment sequencing, adverse effects, and rebound bone loss after denosumab discontinuation remain important limitations. Bone- and cell-targeted nanoparticle delivery may therefore provide an adjunctive strategy for selected high-risk patients by enabling sustained modulation of bone-remodeling pathways with reduced off-target exposure.

### 5.2 Disease-specific delivery challenges for osteoporosis

The specific delivery of OP faces a core challenge: first, the systemic characteristics of the disease. OP affects the bone tissue of the whole body, and the uniform delivery of gene drugs in the bones of the whole body must be achieved. However, existing systemic delivery strategies still face the challenge of rapid clearance mediated by mononuclear phagocyte systems and active and passive localization in the circulation, making it difficult to achieve efficient and uniform enrichment of bones throughout the body [109], [110]. The density of the bone microenvironment and relatively low vascularization are other major obstacles that limit the penetration of nanocarriers and make it difficult to reach the lesion site effectively [111].

### 5.3 Nanoparticle-mediated gene therapy for osteoporosis

Many studies have demonstrated the effectiveness of nanoparticles in OP gene therapy, especially in gene suppression therapy (Table 2). Gene therapy for OP can be categorized into in vivo and in vitro treatments. In vivo therapies can be further divided into systemic and local therapies. The main mode of systemic therapy administration is intravenous injection, whereas intramedullary injection, intra-articular injection, and scaffold implantation are the main modes of local therapy administration (Fig. 2).

**Table 2:** Gene delivery systems for OP treatment.

Nucleic Acids	Delivery System	In vivo or vitro	Administration method	Ref.
SOST siRNA and osteostatin	MSNs modified with an ALN functionalized poly(ethyleneglycol) (PEG) ligand	In vivo (OP mouse model)	Tail vein injection	[29]
Sema4d siRNA	A bone-targeting delivery system based on N-(2-hydroxypropyl) methacrylamide (HPMA) copolymers with D-aspartic acid octapeptide (D-Asp8)	In vivo (OP mouse model)	Tail vein injection	[31]
Plekho1 siRNA	Cationic liposomes based on DOTAP and (AspSerSer)6	In vivo (OP mouse model)	Tail vein injection	[5]
SFRP1 GapmeR	Lipid-polymer nanoparticles based on PLGA and DOTAP and functionalized with aptamers	In vivo (OP mouse model)	Tail vein injection	[25]
SIRT1 saRNA	A tetrahedral framework nucleic acid system with saRNA-SIRT1 gene activation functionality (tsa system)	In vivo (Diabetic OP mouse model)	Tail vein injection	[32]
SOST siRNA and osteostatin	MSNs coated with poly ethylenimine	In vivo (OP mouse models)	Intramedullary injection	[30]
RANKL-CRISPR/Cas9 plasmid	An acid responsive metal-organic framework (ZIF8-NaHCO3@Cas9)	In vivo (OP mouse model)	Intramedullary injection	[27]
GAS5	LNP delivery system using the novel ionizable lipids	In vivo (Rat with OP and rotator cuff tear)	Intraarticular injection	[6]
Ckip-1 siRNA	Chitosan/siCkip-1 biofunctionalized titanium	In vivo (OP Rat model)	Through local implantation	[17]
BCL2L2 siRNA and TRIB2 siRNA	TransIT-TKO/siRNA	In vitro (MSCs)	Through local implantation	[26]

**Fig. 2:**
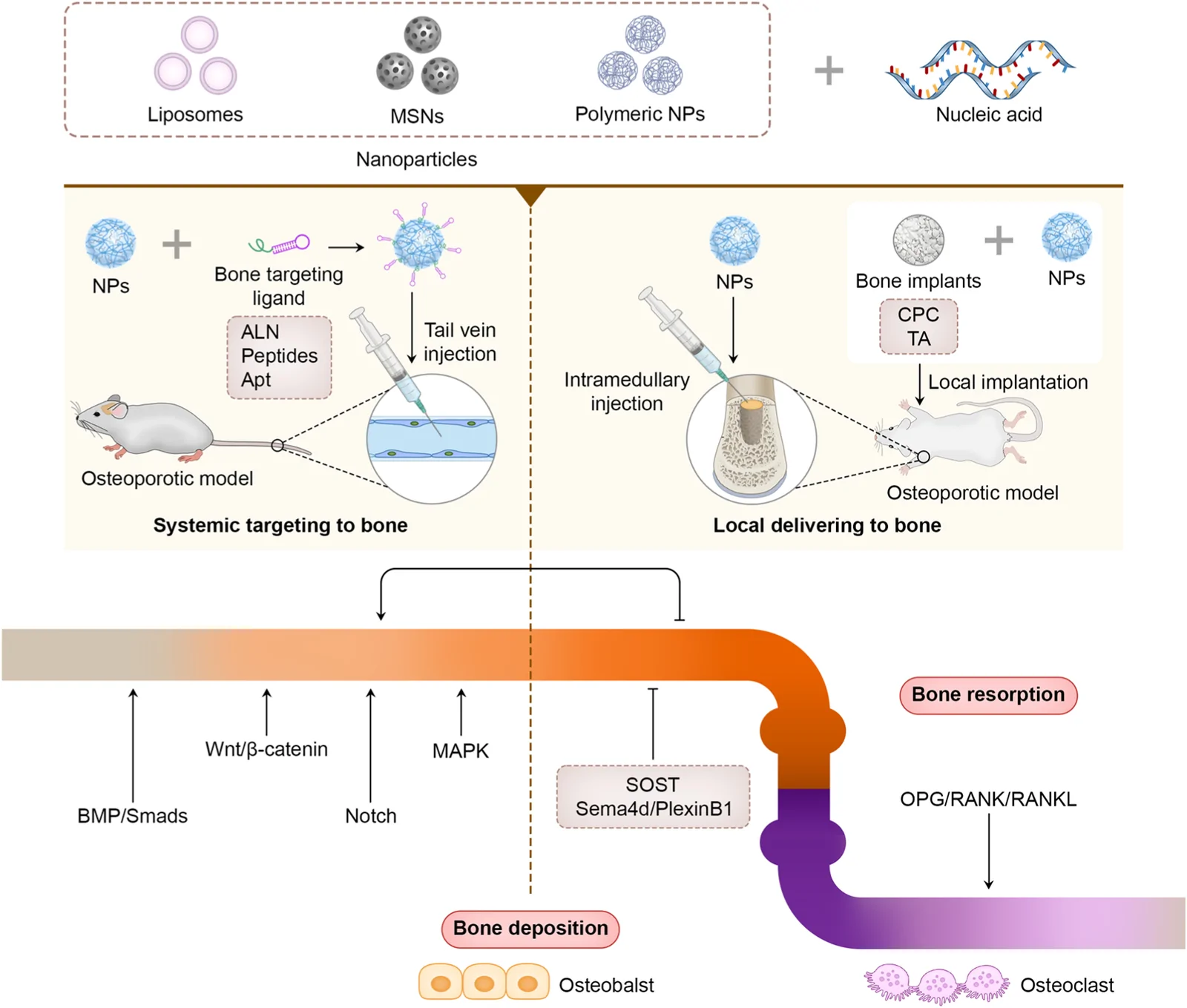
Delivery strategies and implications of nanoparticle delivery system-mediated gene therapy for OP. Nanoparticles used for osteoporosis gene therapy mainly include liposomes, polymeric nanoparticles, and inorganic nanoparticles (MSNs). Their nucleic acid drug delivery strategies include systemic drug delivery (active targeting through modification of ALN, peptides, or aptamers) and local drug delivery (including intramedullary cavity injection, implantation combined with implants, and slow release). Mechanism of action of gene delivery: ① gene addition strategy (delivering DNA of positive regulatory proteins of bone formation pathway, acting on osteoblasts); ② gene suppression strategy (delivering siRNA of negative regulatory protein of bone formation pathway or siRNA of positive regulatory protein of bone resorption pathway, acting on osteoblasts and osteoclasts respectively).

#### 5.3.1 Systemic targeted delivery of in vivo gene therapy

Since OP is a systemic disease, systemic delivery of gene therapy drugs may be preferable. Systemic delivery of nucleic acid drugs requires the delivery system that is specific to the bone site. Therefore, many studies have focused on the surface modification of nanoparticles to improve their targeting ability. Bisphosphonates, oligopeptides, and peptides are several common bone-targeting molecules. Bisphosphonates have a strong affinity for hydroxyapatite (HA) in bone, and Alendronate (ALN) is one of its representatives, which is distributed on bone-forming surfaces and bone-resorbing surfaces. Raimundo et al. used MSNs modified with an ALN functionalized PEG ligand to deliver SOST siRNAs and osteogenic inhibitors, which are a system that protects siRNAs from the enzyme and aggregates around the HA. They successfully improved the bone microstructure in osteoporotic mice after three weeks of systemic treatment [29]. The eight repeats of aspartic acid (Asp8), a representative targeting oligopeptide, have a stronger affinity for the highly crystalline hydroxyapatite of osteoclast-covered bone resorption surfaces and are therefore primarily targeted to bone resorption surfaces. Zhang et al. developed a bone-targeting delivery system based on N-(2-hydroxypropyl) methacrylamide (HPMA) copolymers with D-aspartic acid octapeptide (D-Asp8), loaded with sema4D siRNA, and delivered it to osteoporotic mice by tail vein injection. The results showed that the bone-targeting system had a stronger ability to specifically distribute to bone tissues. It dramatically increased osteoclast coverage and bone mineral deposition on the bone surface, as compared to delivery of sema4D siRNA only [112]. Similarly, Zhang et al. attached (AspSerSer)6, a peptide that targets the surface of bone formation, to DOTAP-based cationic liposomes and obtained good OP therapeutic effects in mouse models [5]. Aptamers are small, single-stranded nucleic acid molecules or peptides that are often used as targeting ligands for the diagnosis and treatment of diseases. García et al. found that when cells were treated with LNPs containing SFRP1 GapmeR and functionalized with aptamers, a greater reduction in SFRP1 expression was observed. In vivo experiments in mouse models showed that such aptamer-functionalized LNPs had greater bone tissue targeting ability [25].

Collectively, these studies demonstrate that bone affinity is not equivalent to cell-specific delivery. ALN, D-Asp8, and (AspSerSer)6 target different bone surfaces [5], [29], [112], whereas aptamers may provide additional molecular selectivity [25]. More recent systems have further incorporated osteoimmune regulation or microenvironment-responsive CRISPR/Cas9 delivery [27], [32], indicating a shift from simple bone accumulation toward integrated targeting and disease modulation. Nevertheless, cell-specific biodistribution, durability of gene regulation, and repeat-dose safety require further investigation.

When OP gene therapy is administered systemically, in addition to targeting bone tissue, nanoparticles can also act on immune cells to remodel the bone microenvironment, indirectly inhibit bone resorption, and promote bone formation by regulating the interaction between immune cell function and bone metabolism. For example, Cai et al. designed a novel framework nucleotide nano-system "Tsa" with target gene activation capability, consisting of a tetrahedral nucleic acid scaffold shell and saRNA targeting SIRT1 activation. This in vivo experiment in diabetic OP mouse showed that Tsa regulates the innate immune system by upregulating SIRT1 expression and promotes M2 macrophage polarization. Through M2 macrophage-osteoblast interactions, Tsa restores the bone immune microenvironment, thereby treating diabetic osteoporosis [32].

#### 5.3.2 Local delivery of in vivo gene therapy

Localized osteoinduction in vivo delivers therapeutic genes directly to a specific site in the bone, requires less vector targeting, reduces the impact on other tissues, and is suitable for specific high-risk fracture areas. Localized therapy can be achieved by injecting drugs into the bone marrow cavity. Raimundo et al. surface-modified MSNs with PEIs to improve their biocompatibility. They then used this carrier to deliver SOST siRNA and osteoclast inhibitor via bone marrow injection in the mouse femur for the treatment of OP. The results of the study showed that the system demonstrated high silencing of SOST and high levels of osteogenic ability in vitro and in vivo, and that the co-administration of SOST siRNA and osteoclast inhibitor produced a synergistic effect [30]. In addition, Lin et al. developed an acid-responsive metal-organic framework for the delivery of RANKL-CRISPR/Cas9 plasmid to osteoporotic mice via intramedullary injection and demonstrated the effectiveness of this method. In addition to intramedullary injection, in vivo studies in rats by Li et al. have shown that intra-articular injection is also an effective method for local delivery [6].

In addition, the use of nanocarriers in combination with bone implants for gene delivery could provide mechanical support to synergistically promote bone regeneration, while further modulating the release rate. Wang et al. reported a localized delivery system targeting osteoclasts. The system encapsulated siRNA in degradable PLGA and incorporated it into an injectable calcium phosphate cement (CPC). These particles can only be taken up by osteoclasts and their phagocytic precursors at the implantation site through phagocytosis. In vitro experiments have shown that they are dispersed in CPC and can be gradually released with the CPC degradation, thus realizing a controlled release strategy for gene therapy drugs [113]. Zhang et al. promoted adsorptive binding of chitosan/siCkip-1 complexes to peptide implants by thermal alkali (TA) treatment to form TA-chitosan/siCkip-1. TA-chitosan/siCkip-1 improved the drawbacks of chitosan/siR nanoparticles due to the wide distribution and irregular morphology of chitosan molecules, and high efficiency of siRNA delivery and high knockdown efficiency were observed in both in vitro and in vivo delivery to rats [17].

#### 5.3.3 In vitro gene therapy

OP can also be treated with in vitro gene therapy using nanoparticles. Cells such as MSC or ASDCs are genetically modified in vitro using nanocarriers, and the modified cells are then introduced in vivo. Andersen et al. designed a siRNA delivery system that successfully knocked down BCL2L2 and TRIB2 in human MSCs cultured in vitro. These cells were implanted into a mouse model, and enhanced adipogenesis and osteoblast differentiation were observed eight weeks after implantation [26].

### 5.4 Effectiveness, shortcomings, and optimization

The core reason for the current success of existing strategies lies in the rational use of the structural advantages of nanocarriers. Nanoscale size can help gene drugs cross part of the biological barrier, surface modification technology can achieve targeted improvement, biocompatibility of vectors can reduce immune rejection, and gene drugs can directly regulate bone metabolism-related genes and signaling pathways, intervening in the process of OP at the etiological level.

Although these strategies have shown promising success in disease research, bone-targeted nanoparticle delivery systems have rarely been successfully translated into clinical applications. This is limited by several factors: insufficient targeting to specific cells or molecules, the trade-off between drug loading stability and drug release, poor blood circulation, and issues related to biosafety and immunogenicity [114], [115].

To improve the bone targeting of nanoparticles, future research should focus on the development of nano-systems with dual or multiple targeting mechanisms. For example, combining traditional bone-targeting molecules with cell-specific ligands further improves the enrichment efficiency of drugs in specific target cells and achieves more accurate cell-specific delivery.

Ensuring that nanoparticles remain stable before reaching the target while achieving controllable and efficient gene release at the target is a key challenge. Intelligently responsive nanocarriers can be designed to respond to the pathological microenvironment of OP, such as inflammation, low pH, or specific enzymes [114].

Challenges associated with blood circulation during systemic administration and the immunogenicity of nanomaterials are also important concerns. To this end, biomimetic strategies such as cell membrane-coated nanoparticles (CMNPs) can be further developed to actively evade immune recognition and clearance by simulating the surface characteristics of host cells, thereby effectively extending the cycling time of nanoparticles in vivo and simultaneously improving their biocompatibility and delivery stability [116].

In addition, bone remodeling is closely related to the immune system, and immune cells in the bone marrow microenvironment have an important impact on bone metabolism. However, most existing research on nanoparticle gene therapy focuses only on the regulation of bone metabolic pathways. Future research should delve into the effectiveness of gene therapy strategies targeting the bone immune microenvironment (e.g., Treg/Th17 balance and macrophage polarization), which will help design more comprehensive and effective nanoparticle-based gene therapy strategies for OP [117].

## 6 Osteoarthritis: Cartilage protection and intra-articular delivery

### 6.1 Pathogenesis and current status of osteoarthritis treatment

Osteoarthritis (OA) is a common degenerative arthritis and a leading cause of disability in the elderly population. Aging, hormonal imbalances, and other factors lead to impaired penetration of nutrients and accumulation of metabolic wastes in the joints, ultimately leading to degradation of articular cartilage, synovial inflammation, and subchondral osteosclerosis [118]. Protein-hydrolyzing enzymes, pro-inflammatory cytokines, and reactive oxygen species are major factors contributing to OA development [119], [120]. Current OA management is centred on exercise, weight management, topical or oral nonsteroidal anti-inflammatory drugs, and short-term intra-articular corticosteroid injections, with joint replacement reserved for advanced disease [121]. These treatments mainly improve pain and function, whereas no approved pharmacological therapy reliably restores cartilage or prevents whole-joint structural progression. Pathology-responsive intra-articular nanoparticles may improve local retention and enable targeted delivery of nucleic acids to cartilage or synovial cells [122], [123]. Their clinical value should be evaluated using both symptomatic outcomes, such as WOMAC or KOOS, and structural outcomes, such as quantitative magnetic resonance imaging of cartilage. OA should therefore be viewed as a whole-joint disorder in which cartilage damage, synovitis, abnormal subchondral bone remodeling, and immune activation are mutually reinforcing rather than isolated events. Cartilage-derived matrix fragments and danger signals activate synovial fibroblasts and macrophages, whereas cytokines, extracellular vesicles, and altered osteochondral mechanics feed back to chondrocytes and bone cells [124], [125], [126].

### 6.2 Gene therapy mechanisms and delivery challenges for osteoarthritis

Gene therapy has the potential to be an ideal treatment for OA because it can inhibit further development of synovial inflammation and cartilage degradation by blocking these causative factors and related signaling and metabolic pathways before the onset of severe OA. Gene therapy mechanisms for OA primarily target three core aspects: blocking pathogenic pathways, protecting and repairing cartilage, and modulating the inflammatory microenvironment. First, by silencing matrix metalloproteinases (MMPs) or introducing anti-inflammatory genes (such as IL-10, IL-1Ra, and TGF-β), the expression of cartilage matrix-degrading enzymes is suppressed, and the release of pro-inflammatory factors is reduced, thereby slowing or even halting cartilage degeneration [18], [23]. Second, targeting chondrocyte-related genes (e.g., GDF5 and SOX9) effectively promotes chondrocyte formation and regulates the expression of ECM proteins in chondrocytes [8], [19]. Third, gene therapy modulates local metabolic and signaling pathways within the joints. For instance, silencing oxidative stress-related genes (such as p66shc and p47phox) helps remove reactive oxygen species, mitigating oxidative damage to cartilage [10], [11]. Simultaneously, it regulates abnormally activated signaling pathways like NF-κB, contributing to the restoration of homeostasis in the joint microenvironment [34].

However, it is important to note that cartilage tissue is mainly composed of a large amount of dense ECM, and chondrocytes are dispersed in stromal pits, with extremely low cell density and lack of blood vessels, which brings unique challenges to the delivery, cell uptake, and function of gene therapy drugs [127], [128]. Beyond cartilage penetration, delivery systems must also overcome rapid synovial clearance, heterogeneous uptake by fibroblast-like synoviocytes and immune cells, and limited access to subchondral bone. Because the dominant cell populations and remodeling states vary with disease stage, spatially and temporally controlled delivery is required across the whole-joint unit.

### 6.3 Nanoparticle-mediated gene therapy across the whole-joint unit

Gene therapy for OA can be implemented in vivo or ex vivo. Unlike OP, in vivo gene therapy for OA is usually delivered locally by intra-articular injection. Nanoparticles can be engineered through size, surface charge, hydrophilicity, and ligand modification to reach cartilage, synovium, or selected cell populations (Fig. 3). To reflect OA as a whole-joint disease, the evidence below is organized by the cartilage-synovium-subchondral bone-immune cell unit, while retaining the distinction between passive and active targeting and between in vivo and ex vivo strategies (Table 3).

**Fig. 3:**
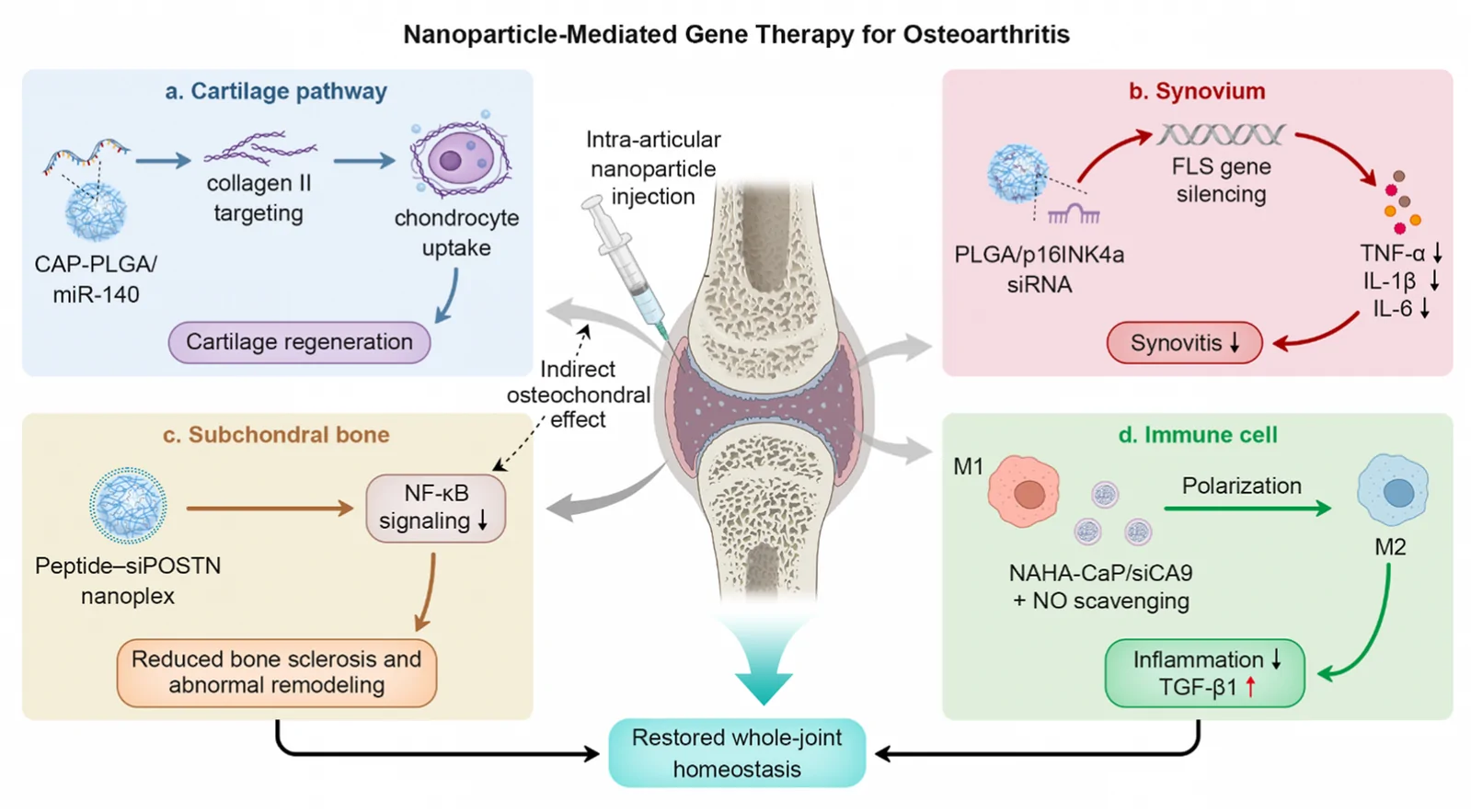
Representative nanoparticle-mediated gene therapies for osteoarthritis. Intra-articular nanoparticles restore whole-joint homeostasis through tissue- and cell-specific mechanisms: (a) CAP-PLGA/miR-140 nanoparticles target cartilage and promote regeneration; (b) PLGA/p16INK4a siRNA nanoparticles silence genes in fibroblast-like synoviocytes and alleviate synovitis; (c) peptide–siPOSTN nanoplexes suppress NF-κB signaling and reduce abnormal subchondral bone remodeling; and (d) NAHA-CaP/siCA9 nanoparticles promote M1-to-M2 macrophage polarization and suppress inflammation. CAP, cartilage-targeting peptide; PLGA, poly(lactic-co-glycolic acid); POSTN, periostin; CA9, carbonic anhydrase IX.

**Table 3:** Gene delivery systems for OA treatment across the whole-joint unit.

Nucleic Acids	Target site	Delivery System	In vivo or vitro	Administration method	Ref.
miR-124	Cartilage	A tetrahedral framework nucleic acid (tFNA) system	In vivo (OA rat model)	Intra-articular injection	[33]
NF-κB siRNA	Cartilage	A tetrahedral framework nucleic acid (tFNA) system	In vivo (rats with temporomandibular joint OA)	Intra-articular injection	[34]
IL-1Ra and IL-10 pDNA	Cartilage	Chitosan–DNA nanoparticles	In vivo (OA rabbit model)	Intra-articular injection	[18]
Ihh siRNA	Cartilage	LNPs based on Dlin-KC2-DMA	In vivo (OA rat model)	Intra-articular injection	[7]
HIF-2α siRNA and curcumin	Cartilage	MIL-101-NH2 nanoparticles	In vivo (OA mouse model)	Intra-articular injection	[21]
GDF-5 pDNA	Cartilage	Nano-microspheres containing chitosan, hyaluronic acid, and chondroitin sulfate	In vivo (OA rabbit model)	Intra-articular injection	[19]
p66shc siRNA	Cartilage	Poly (lactic-co-glycolic acid) (PLGA)-based nanoparticles	In vivo (OA rat model)	Intra-articular injection	[10]
p47phox siRNA	Cartilage	Poly (lactic-co-glycolic acid) (PLGA)-based nanoparticles	In vivo (OA rat model)	Intra-articular injection	[11]
miR-140	Cartilage	Polyvinylamine (PVAm) modified by poly lactic-co-glycolic acid (PLGA) and cartilage-targeting peptide (CAP)	In vivo (OA mouse model)	Intra-articular injection	[12]
SOX5 and SOX9 mRNA	Cartilage	Rationally optimized SM-102-based LNPs	In vivo (OA rat model)	Intra-articular injection	[8]
miR-224-5p	Synovium	Amino-acid-modified PAMAM nanoparticles	In vivo (OA mouse; cartilage and synovium)	Intra-articular injection	[35]
p16INK4a siRNA	Synovium	PLGA nanoparticles	In vivo (pMMx mouse; synovial FLSs)	Intra-articular injection	[36]
Periostin siRNA	Subchondral bone/osteochondral unit	Peptide-siRNA nanoplex	In vivo (DMM mouse; osteochondral outcomes)	Intra-articular injection	[37]
rhFGF18 mRNA	Subchondral bone/osteochondral unit	WG-PL14 lipid nanoparticles	In vivo (OA mouse; cartilage and subchondral bone)	Intra-articular injection	[38]
LEPR shRNA	Immune cells/intercellular crosstalk	Macrophage-membrane-coated HA/PEI nanoparticles	In vivo (OA model; macrophages and synoviocytes)	Intra-articular injection	[39]
CA9 siRNA	Immune cells/intercellular crosstalk	NAHA-CaP/siRNA nanoparticles	In vivo (OA mouse and rat; synovial macrophages)	Intra-articular injection	[40]
CDKN1A pDNA	Ex vivo engineered-cell therapy	Copper sulfide (CuS) nanoparticles	In vitro (MSCs)	Intra-articular injection	[22]
TGF-β1 plasmid	Ex vivo engineered-cell therapy	Copper sulfide@phosphatidylcholine (CuS@PC) nanoparticles	In vitro (MSCs)	Intra-articular injection	[23]
ANGPTL4 shRNA and SOX duo (SOX-9, -6)	Ex vivo engineered-cell therapy	Dexamethasone-conjugated polyethyleneimine (DEXPEI) nanoparticles	In vitro (ADSCs)	Intra-articular injection	[20]

#### 6.3.1 Cartilage-directed gene delivery

For cartilage-directed delivery, passive targeting relies on the inherent properties of nanoparticles (e.g., size, surface charge, and hydrophilicity) and the physiological characteristics of the diseased tissue, allowing enrichment without additional modifications. Chitosan-, PLGA-, tetrahedral nucleic acid-, lipid-, and metal-organic framework-based systems can use size, charge, matrix affinity, degradability, and microenvironment responsiveness to penetrate cartilage. Active ligands such as hyaluronic acid (HA), chondroitin sulfate, and cartilage-targeting peptides further increase cartilage or chondrocyte uptake.

Chitosan is one of the earliest and most widely used nanocarriers for gene therapy and drug delivery in OA treatment. Its passive targeting of chondrocytes may be achieved through electrostatic adsorption on cartilage surfaces. It has a high cationic charge, size fitness, strong bioadhesion, and response to enzymes such as MMPs. Zhang et al. injected chitosan-DNA nanoparticles containing IL-1Ra or IL-10 into the knee joints of OA rabbits, and the results showed that the application of chitosan-IL-1Ra nanoparticles reduced cartilage degradation and delayed the progression of structural changes in OA [18]. Although chitosan has many advantages, its relatively poor targeting ability and low transfection level have made its clinical use challenging. Therefore, some studies have started to look for various strategies, such as combining with other biopolymers, to improve its targeting and transfection ability. Lu et al. copolymerized chitosan with low-molecular-weight PEI and developed chitosan-polyethyleneimine (CP) nanoparticles as a novel non-viral gene vector for OA gene therapy. In vitro experiments showed that the transfection efficiency of this vector in chondrocytes and synoviocytes was significantly higher than that of CS/DNA and PEI/DNA nanoparticles [129].

The mucus layer covering the surface of articular cartilage is rich in mucin networks, creating a natural size screening barrier. It has been shown that only nanoparticles smaller than 200 nm can easily pass through mucus without being immobilized by the natural size-filtering mechanism. PLGA nanoparticles can be designed below 100 nm [11], which can easily penetrate the cartilage matrix and diffuse into the deeper cartilage layers. In addition, PLGA nanoparticles enable passive targeting of chondrocytes through their degradability, PH responsiveness, and encapsulation in positively charged materials (e.g., PEI). Several studies have demonstrated the efficacy and safety of PLGA-based nanoparticles for in vivo delivery of nucleic acid drugs in OA treatment. Shin et al. demonstrated that PLGA-based nanoparticles delivering p66shc siRNA and p47phox siRNA improved MIA-induced pain behavior in the knee joints of OA rats, as well as cartilage damage, inflammatory response, and ROS production [10], [11].

The tetrahedral nucleic acid framework is 10-20 nm in size, which is much smaller than the pore size of the cartilage matrix, allowing rapid diffusion into the deeper layers of cartilage. Liao et al. utilized a tetrahedral nucleic acid framework system loaded with NF-κB siRNAs and administered intraarticularly in temporomandibular joint osteoarthritis (TMJOA) model rats. The results demonstrated that this delivery system, compared to the use of siRNAs alone, showed better NF-κB silencing effects and upregulation of NRF2/HO-1 pathway, which more effectively inhibited the release of inflammatory factors, cellular oxidative stress, and apoptosis, and provided significant protection to the cartilage matrix and subchondral bone [34]. In addition, Shi et al. provided MicroRNA-124 through tetrahedral framework nucleic acids to promote cartilage regeneration and ameliorate OA [33].

In addition to their small particle size, the LNPs can bind to hydrophobic regions within the cartilage matrix, thereby enhancing their local accumulation. Meanwhile, LNPs are attracted to negatively charged proteoglycans, establishing a cartilage-specific delivery system [7]. Wang et al. showed that intra-articular injection of LNP-Ihh siRNA can slow down the progression of OA in a rat Anterior Cruciate Ligament Transection (ACLT) OA model [7]. Through systematic formulation screening, Yu et al. identified the LNP combination that most effectively targeted chondrocytes and maximized intracellular mRNA delivery. In a rat OA model, the therapeutic effect of this LNP delivering SOX5 and SOX9 mRNA was verified [8].

Zhang et al. developed a pH-responsive metal-organic framework material (MIL-101-NH2 NPs) with effective pH-responsiveness and biodegradability, and proved that that the co-delivery of CCM and siHIF-2α via this nanomaterial has a synergistic effect on OA mouse model, slowing down cartilage matrix depletion in the development of OA more effectively than other treatment groups [21].

Chondroitin sulfate, a negatively charged glycosaminoglycan present in natural cartilage tissues, can also be recognized by CD44, enabling active targeting of gene therapy vectors for OA [130]. Moghadam et al. used chitosan-chondroitin sulfate nanoparticles to load MMP13 siRNA and introduced them into chondrocytes in vitro. They found that this new nanoparticle had higher cellular uptake efficiency than chitosan nanoparticles and liposome nanoparticles [131]. Chen et al. constructed a novel non-viral vector using chitosan, HA, and chondroitin sulfate and delivered growth factor-5 (GDF-5) to in vitro-cultivated chondrocytes and rabbit model of OA. The results showed that the expression of the ECM protein was markedly increased in chondrocytes, and the cartilage morphology and joint structure of OA animals were significantly improved [19].

Cartilage-targeting peptide (CAP) is a specific binding peptide of type II collagen, a major component of the cartilage matrix. Zhao et al. used it as a targeting ligand to design an RNA delivery platform for OA treatment, named CAP-PVAm-PLGA/r-miR-140 nanoparticles (CPP-NPs), which have high permeability and cartilage-targeting properties. After intra-articular injection of CPP-NPs, the therapeutic amount of nucleic acid drugs was effectively maintained, and the cartilage degradation area and synovial inflammation score of OA mouse were significantly restored [12].

#### 6.3.2 Synovium-directed gene delivery

The synovium is readily exposed after intra-articular injection and contains fibroblast-like synoviocytes (FLSs) and resident or infiltrating immune cells that sustain cytokine and protease production. Synovium-directed delivery can therefore interrupt the upstream inflammatory input to cartilage rather than treating cartilage degradation alone. Ligands such as HA are particularly relevant because CD44 is expressed on OA synovial cells.

Hyaluronic acid (HA) is one of the most commonly used ligands. Fragments of HA or low-molecular-weight HA can bind to various cellular receptors highly expressed on the surface of tissue cells in OA, such as CD44 or TLR-4 [132], [133]. Several research groups have demonstrated that HA binding to nanoparticles may increase their uptake in the disease state of OA. In an in vitro study, Lu et al. utilized HA/chitosan nanoparticles to deliver exogenous genes into primary chondrocytes. Under the same conditions, the transfection efficiency of HA/chitosan-pDNA nanoparticles was significantly higher than that of chitosan-pDNA nanoparticles, and the average cell viability of more than 90% proved the safety of this delivery vector [134]. Moreover, in vitro experiments conducted by Qiu et al. demonstrated that HA/CS nanoparticles also exhibit targeting ability towards synovial cells [135]. Further in vivo experiments demonstrated that HA/CS-CrmA nanoparticles significantly attenuated cartilage damage and loss of type II collagen in cartilage in the rat ACLT model of OA and significantly inhibited synovial inflammation and the expression of IL-1β, MMP-3, and MMP-13 in synovial membranes [136].

Direct evidence for FLS-directed RNA interference was provided by Park et al., who encapsulated p16INK4a siRNA in PLGA nanoparticles. After intra-articular administration, fluorescent nanoparticles accumulated mainly in the synovium; p16INK4a knockdown reduced TNF-α, IL-1β, and IL-6 in FLSs, relieved pain, and secondarily decreased MMP13, collagen X, and NITEGE in cartilage in a partial meniscectomy model [36].

A broader dual-compartment strategy was demonstrated by Chen et al. using amino-acid-modified polyamidoamine nanoparticles to deliver miR-224-5p. The formulation inhibited inflammatory hyperplasia of FLSs while restoring chondrocyte autophagy and matrix anabolism, and it reduced synovial hypertrophy, osteophyte formation, and subchondral bone sclerosis in OA mice [35]. These studies show that synovial targeting can protect cartilage through modification of tissue crosstalk.

Complementing these nanogene studies, Jia et al. used single-cell transcriptomics and adenoviral gain- and loss-of-function experiments to show that PPARγ regulates ESCRT-dependent exosome biogenesis in FLSs and modifies exosomal ANXA1/FPR2 signaling to protect chondrocytes [137]. This study identifies a tractable synovium-to-cartilage communication axis that could be targeted by future nucleic-acid delivery systems.

#### 6.3.3 Subchondral bone remodeling and osteochondral crosstalk

Subchondral bone is a dynamic therapeutic compartment. Early OA is associated with increased osteoclast-mediated resorption, angiogenesis, and bone marrow lesions, whereas later disease often shows excessive osteoblast activity and sclerosis; these stage-dependent changes alter mechanical load transfer and soluble signaling to cartilage [124], [125]. However, OA nanogene studies that directly target osteoclasts or osteoblasts remain scarce, and most current evidence reports subchondral outcomes secondary to intra-articular delivery.

Duan et al. used a peptide-siRNA nanoplex to silence periostin after destabilization of the medial meniscus. In addition to reducing cartilage damage and NF-κB-driven catabolism, treatment decreased subchondral bone sclerosis, bone volume fraction, volumetric bone mineral density, and heterotopic ossification [37]. More recently, joint-localized WG-PL14 lipid nanoparticles delivering recombinant human FGF18 mRNA promoted chondrocyte matrix synthesis and remodeled subchondral bone homeostasis in a mouse OA model [38]. Together with the cartilage and subchondral bone protection observed after tetrahedral framework nucleic acid-mediated NF-κB siRNA delivery [34], these data support osteochondral-unit evaluation while also highlighting the need for bone-cell-specific delivery.

#### 6.3.4 Immune-cell modulation and intercellular crosstalk

Immune cells, especially synovial macrophages, connect cartilage catabolism, synovitis, and bone remodeling through IL-1β, TNF-α, IL-6, reactive oxygen and nitrogen species, and osteoclastogenic signals. Yan et al. developed NAHA-CaP nanoparticles that combined carbonic anhydrase IX (CA9) siRNA with nitric oxide scavenging. The system reduced CA9 mRNA by approximately 90% in macrophages, shifted M1 cells toward an M2 phenotype, increased TGF-β1 expression, and protected cartilage across early and late OA models [40].

Zhou et al. condensed shRNA against the leptin receptor (LEPR) and the anti-inflammatory peptide KAFAK with polyethyleneimine, added HA, and coated the complex with an activated macrophage membrane. The biomimetic system suppressed pro-inflammatory responses in macrophages and synoviocytes and reduced synovitis and joint damage [39]. Intercellular communication also provides gene targets: osteoclast-derived apoptotic bodies accumulate with OA severity and promote aberrant subchondral bone formation through RANKL reverse signaling, while rapamycin partially counteracts this pathway [138]. Pyroptosis-related signaling provides another joint-wide feedback mechanism among macrophages, FLSs, and chondrocytes [126]. Thus, future nanogene systems should be assessed not only for uptake in a single cell type but also for their ability to break pathogenic feedback loops across the whole joint.

#### 6.3.5 In vitro gene therapy

In addition to in vivo gene therapy, several studies have demonstrated the efficacy of certain nanoparticles for in vitro gene therapy of OA. Because engineered MSCs and ADSCs act through both cell replacement and paracrine immunomodulation, their effects should be evaluated in cartilage, synovium, immune-cell populations, and subchondral bone rather than by cartilage histology alone.

Liu et al. and Cai et al. introduced therapeutic genes (CDKN1A or TGF-β1) into MSCs using copper sulfide (CuS) nanoparticles, and OA mouse models were treated with intra-articular injections of these edited MSCs. The results showed that the operation could effectively regulate fibroblast activity and promote cartilage repair, whereas pure MSCs had a limited effect on cartilage regeneration in DMM surgery-induced OA mice [22], [23]. Jeong et al. designed dexamethasone-conjugated polyethyleneimine (DEXPEI) nanoparticles loaded with SOX duo (SOX-9, -6) and ANGPTL4 shRNA and transfected them into ADSCs. These modified ADSCs had enhanced cartilage formation and reduced levels of cyclooxygenase (COX-2) and MMP13 in synovial fluid of a rat model of OA [20].

### 6.4 Effectiveness, shortcomings, and optimization

Nanoparticle-mediated gene therapy for OA has demonstrated remarkable efficacy, primarily because therapeutic genes can be enriched in defined joint compartments or cell populations through passive or active targeting. Simultaneously, nanoparticles enhance nucleic-acid stability and transfection efficiency and can reduce systemic exposure, thereby contributing to delayed OA progression. The most informative studies now move beyond cartilage histology and assess synovitis, immune-cell phenotype, and subchondral bone structure together.

Although many studies have confirmed the therapeutic potential of nanoparticles in different animal models, their translation into clinical applications remains limited. Current major challenges include limited drug-loading capacity, premature burst release, and short in vivo retention time [139]. To address these issues, the development of hybrid nanomedicine systems, such as hydrogel microsphere-based delivery platforms, should be explored to prolong intra-articular retention and reduce dosing frequency [140]. Co-modification of nanoparticles with cartilage-targeting molecules and environmentally responsive molecules, such as reactive oxygen species, can synergistically enhance their targeting capability toward cartilage tissue and achieve controlled drug release [141]. The retention of nanomaterials is influenced not only by their intrinsic properties but also by the physiological microenvironments where they are localized [142]. However, the structural and physiological changes during OA progression often impose dynamic and stage-specific demands on nanoparticle properties, such as size and surface charge. Currently, studies on the adaptability between different stages of OA and nanoparticle characteristics remain insufficient. Future work should focus on systematic investigations to address this research gap. OA is a multifactorial disease; however, few nanoparticle delivery systems have been developed for gene therapy targeting its multiple pathological processes. For example, although Zhang et al. successfully achieved co-delivery of two drugs, their study was not designed to specifically address distinct pathological pathways [21]. Future efforts should focus on developing of multimodal delivery systems. Furthermore, nanomaterials can be extended for drug delivery to other joint tissues, such as the fat pad, meniscus, ligaments, and subchondral bone, thereby enabling more systematic and comprehensive therapeutic delivery strategies [143]. Accordingly, future delivery systems should be benchmarked using a whole-joint panel that includes cartilage matrix integrity, synovial cell states, macrophage polarization, osteoclast and osteoblast remodeling, angiogenesis, and intercellular signaling. Compartment-specific biodistribution and stage-dependent efficacy should also be reported to distinguish a true whole-joint intervention from a cartilage-dominant effect.

## 7 Intervertebral disc degeneration: navigating the avascular niche with active targeting and staged-release nanocarriers

### 7.1 Pathogenesis and current status of intervertebral disc degeneration treatment

Intervertebral disc degeneration (IDD) is frequently detected on magnetic resonance imaging in asymptomatic individuals and should not be considered equivalent to symptomatic discogenic low-back pain [144]. Current treatment, including exercise-based rehabilitation, nonsteroidal anti-inflammatory drugs, and selected surgical procedures, primarily improves pain and function but does not restore disc cellularity or extracellular matrix integrity [145]. For nanoparticle-mediated gene therapy, the most plausible target population comprises patients with persistent discogenic pain and early-to-moderate degeneration after failure of conservative treatment. Local intradiscal delivery may enable sustained modulation of oxidative stress, inflammation, and matrix catabolism [146], but clinical evaluation should combine pain and Oswestry Disability Index outcomes with quantitative imaging and procedural safety.

### 7.2 Gene therapy mechanisms and delivery challenges for intervertebral disc degeneration

Gene therapy is emerging as a novel approach for IDD treatment. The main strategies include (1) regulating the metabolism of the ECM to promote matrix regeneration and (2) targeting the biological factors that promote degeneration, such as anti-inflammatory, antioxidant, anti-fibrotic, and anti-apoptotic factors, to delay degeneration and promote the return of the intervertebral disc (IVD) to its healthy state (Fig. 4). Nanoparticles are more amenable to chemical modification and can mimic the nanofibrous structure of the ECM, thereby promoting tissue regeneration. Thus, they have emerged as an attractive vector for IDD gene therapy. Related research focuses on gene suppression therapy.

**Fig. 4:**
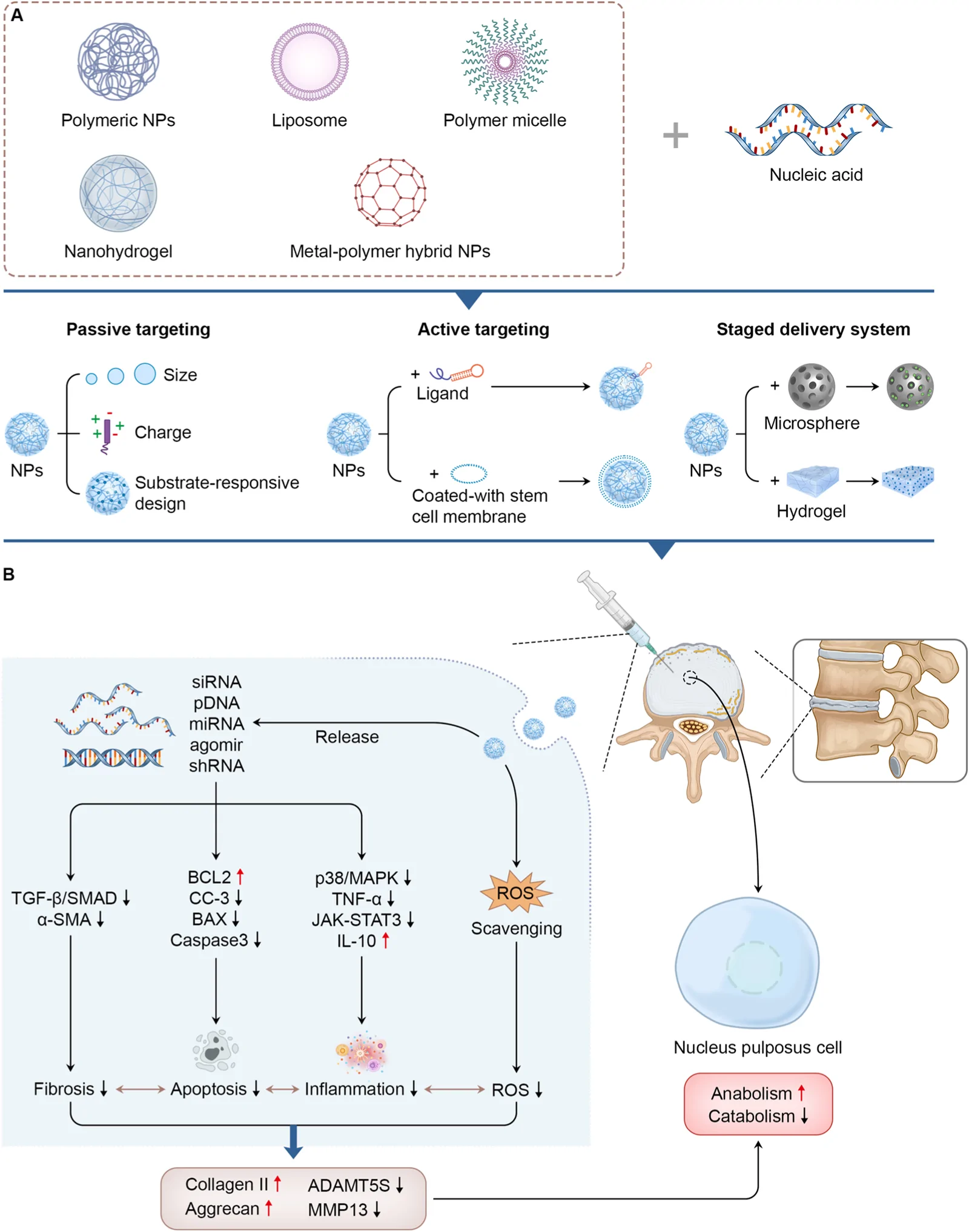
Delivery strategies and implications of nanoparticle delivery system-mediated gene therapy for IDD. (A) Delivery strategies. Nanoparticle types: liposomes, polymer nanoparticles, micelles, nanohydrogels, and metal-polymer hybrid nanoparticles. Delivery strategies: passive targeting (regulation of size, surface charge, and environmental responsiveness), active targeting (surface modification ligand or stem cell membrane encapsulation), and phased release (delivery of nucleic acids into cells after binding to microspheres/hydrogels or releasing nanoparticles by MMPs/pH response). (B) The implications on IDD. Mode of administration: local injection, acting on nucleus pulposus cells. Mechanism of action: Regulate target genes to attenuate fibrosis, apoptosis, and inflammation of nucleus pulposus cells, and some nanoparticles scavenge reactive oxygen species. The final treatment promotes the synthesis of type II collagen and ACAN, reduces ADAMTS5 and MMP13 levels, and treats IDD through balanced metabolism.

NPCs are the main cells targeted by IDD gene therapy, and their apoptosis and metabolic imbalance are considered to be one of the molecular mechanisms associated with IDD [147]. The nucleus pulposus tissue is located in the center of the IVD with abundant ECM (especially proteoglycan and collagen) and is in an environment of low blood supply and high osmotic pressure [148]. Such an environment may affect the diffusion and retention of nanoparticles. Therefore, strategies are needed to enhance the targeting of nanoparticles to NPCs, including passive targeting, active targeting, and phased and intelligent responsive design ( Table 4)(Fig. 4).

**Table 4:** Gene delivery systems for IDD treatment.

Cargo	Delivery System	In vivo or vitro	Administration method	Ref.
Caspase 3 siRNA and ADAMTS5 siRNA	Liposomes	In vivo (rabbit disc degeneration model)	Inject locally into the nucleus pulposus	[9]
Agomir874	Polyethylene glycol (PEG)‐hydrogel	In vivo (rat disc degeneration model)	Inject locally into the nucleus pulposus	[41]
shRNA targeting the leptin receptor (LEPR)	Stem cell membrane-camouflaged (Zeolitic imidazolate frameworks-8) ZIF-8 nanoparticles	In vivo (rat disc degeneration model)	Inject locally into the nucleus pulposus	[28]
miR-150-5p	NPC-specific aptamer-decorated polymeric nanoparticles	In vivo (mouse disc degeneration model)	Inject locally into the nucleus pulposus	[42]
miR-22-3p	A nanoparticle loaded with a specific aptamer (PLGA-PEI-PEG-RD4)	In vivo (mouse disc degeneration model)	Inject locally into the nucleus pulposus	[13]
NR4A1 pDNA	A novel injectable scaffold composed by PLGA NS containing the NR4A1 pDNA-carrying polyplexes and newly developed nanofibrous spongy microspheres (NF-SMS)(HP/NS/NF-SMS)	In vivo (rat disc degeneration model)	Inject locally into the nucleus pulposus	[43]
Grem1 siRNA	Hyaluronic acid (HA) and dopamine (DA)-synthesized HADA loaded by PBA functionalized hyaluronic acid methacrylamide (HAMA) microspheres (M.S.)	In vivo (rat disc degeneration model)	Inject locally into the nucleus pulposus	[44]
miR-29a	Polyplex micelle-loaded injectable hydrogels	In vivo (rabbit disc degeneration model)	Inject locally into the nucleus pulposus	[45]

### 7.3 Nanoparticle-mediated gene therapy for intervertebral disc degeneration

The mechanism of passive targeting of NPCs by nanoparticles is mainly categorized as follows. (1) Local injection route of administration: Nanoparticles are diffusion-dependent for distribution in the medulla and slowly release drugs to improve cellular uptake. (2) Size effect: The optimal particle size is 20-200 nm. Small particles are more likely to diffuse into the deeper layers of the medulla to reach more cells, but they also need to be large enough to avoid rapid clearance. (3) Surface charge: Positively charged particles tend to bind to negatively charged proteoglycans, thereby enhancing their retention. (4) Substrate-responsive design: PH- or enzyme-responsive materials are used to release drugs in the acidic or highly enzyme-active environment of the degenerating medulla.

Studies have shown that liposomes and nanohydrogels can achieve passive targeting of NPCs through local injection and their physicochemical properties. Banala et al. utilized liposomes to deliver siRNAs, showing that synergistic inhibition of Caspase 3 and ADAMTS5 genes was able to limit the degeneration of experimentally damaged IVD [9]. Chen et al. constructed gene-hydrogel microenvironment in situ by injecting a PEG-hydrogel loaded with Agomir874, which was shown to be able to regulate the metabolic balance of ECM in IVDNPCs in vivo [41].

#### 7.3.1 Active targeting

Active targeting of NPCs can be achieved by surface modification of nanoparticles so that the locally delivered drug accumulates in the target tissues and cells, maximizing the cellular transfection effect and minimizing side effects. Wang et al. used metal-organic nanoparticle to load leptin receptor (LEPR) shRNA, then coated them with stem cell membrane and delivered them to NPCs or injury-induced IDD rat models. Stem cells can interact with damaged cells through the membrane, thereby improving the tissue targeting of nanoparticles, prolonging their circulation time in vivo, and attenuating the elicited immune responses. The results showed that these stem cell membrane-camouflaged nanoparticles exhibited excellent gene transfection efficiency in vitro and significant anti-inflammatory effects in vivo [28]. Jiang et al. and Chen et al. encapsulated polymer nanoparticles with NPC-specific aptamers to deliver miR-150-5p inhibitors or miR-22-3p inhibitors to NPCs or injury-induced IDD mouse models, which greatly inhibited the secretion of senescence-related factors and subsequent NPCs denaturation in vitro and alleviated the progression of IDD in mouse [13], [42].

#### 7.3.2 Staged delivery systems

Most conventional nanoparticles cannot achieve the controlled release of gene therapy drugs. Some researchers have begun to focus on staged delivery systems to achieve sustained or on-demand release mechanisms, which effectively extend the duration of localized therapy and reduce the required drug dosage. Feng et al. encapsulated HP polymers carrying NR4A1 pDNA in PLGA nanospheres (NS) and combined them with newly developed nanofiber sponge microspheres (NF-SMS) to form a new injectable scaffold that enables a two-stage gene delivery strategy: (1) the HP polymer containing NR4A1 pDNA is released from the PLGA NS, and (2) the HP polymer carries the NR4A1 pDNA into the cell. This novel delivery system is biodegradable, biocompatible, capable of releasing the polymer carrier in a time-controlled manner, and has high cellular uptake and gene transfection rates. This delivery system significantly reversed myelofibrosis and promoted IVD regeneration in injury-induced IDD rat models [43]. Guo et al. used Grem1 siRNA interacting with bPEI to synthesize siGrem1 NPs, which were subsequently encapsulated into HA and dopamine (DA)-synthesized HADA (termed as HSGN), and the researchers also engineered the PBA functionalized hyaluronic acid methacrylamide (HAMA) microspheres (M.S.) to dynamically load HSGN. Using this smart microgel system to treat IDD, the microspheres can function as a water-retaining agent, and the inflammatory environment can lead to the responsive release of HSGN; HA binds specifically to CD44 on the NPCs, thus exerting a targeting ability, whereas DA has the ability to scavenge ROS, and Grem1 siRNA is endocytosed by NPCs to attenuate apoptosis. The delivery system designed in this study combines the advantages of targeting, antioxidant and anti-inflammatory properties, and sustained and on-demand release to effectively slow down the progression of IDD [44]. In addition, Feng et al. developed a continuous, bioreactive, two-stage miRNA delivery system (miR-29a/PGPC@HG) consisting of multimeric micelles as miR-29a carriers and injectable hydrogel capsules. This delivery system treated IDD via local injection and inhibited fibrosis in IVD. In the first stage, elevated MMP levels in local fibrosis regions triggered hydrogel degradation, thus localizing the sustained release of polyplex micelles in diseased IVD tissues. In the second stage, the released polyplex micelles were responsive to MMPs for detachment of PEG shells, and subsequently, enhanced cellular uptake and endosomal escape were achieved in NPCs [45]. Recent IDD studies show a progression from simple local retention toward multistage delivery. Aptamer-functionalized particles improve nucleus pulposus cell recognition [13], [42], whereas membrane-coated nanoparticles and responsive hydrogel systems provide biomimetic targeting or staged cargo release [28], [44], [45]. However, these systems cannot be directly compared because they use different cargos, models, and outcome measures, while increasing carrier complexity may hinder reproducibility and scale-up. Moreover, their continued reliance on intradiscal injection means that procedure-induced disc injury remains unresolved.

### 7.4 Effectiveness, shortcomings, and optimization

Current research has achieved significant progress in nanoparticle-mediated gene therapy for intervertebral disc degeneration (IDD) through rational design of nanocarrier structures, precise targeting of IDD pathological mechanisms, and targeted optimization of delivery strategies. However, this field still faces several critical challenges and limitations.

Firstly, as a multifactorial disease involving multiple pathways, IDD cannot be comprehensively regulated by single-gene intervention. Therefore, future efforts should focus on developing multi-gene co-delivery systems or exploring nucleic acid-based therapeutics capable of simultaneously modulating multiple key signaling pathways to enhance overall therapeutic efficacy.

Secondly, a biomimetic targeted delivery system should be constructed to enhance targeting efficiency and biocompatibility. For instance, nanoparticles can be coated with exosome membranes derived from NPCs or stem cell membranes [28], [149]. Alternatively, peptides or aptamers with high affinity for degenerated NPCs or EMCs could be screened and developed to modify nanoparticles. This would endow the carriers with bioaffinity and targeting capability while reducing immune clearance [42].

Finally, in terms of delivery methods, nanoparticles currently mainly rely on intradiscal injection, but the acupuncture operation itself may induce or accelerate disc degeneration. In addition, existing single-injection models often fail to achieve sustained and controlled drug release. Therefore, there is an urgent need to develop new biomaterials that can not only protect therapeutic nucleic acids from degradation but also support their long-term sustained release at the lesion site, thereby promoting clinical translation [42].

## 8 Tendon diseases: anti-adhesion and sustained delivery systems

### 8.1 Pathogenesis and current status of tendon diseases treatment

Tendon is a specialized connective tissue structure composed of collagen and a few cells arranged in parallel, connecting muscle and bone and playing an important role in limb movement [150]. Injured tendons heal spontaneously, and the healing process consists of three main phases: inflammatory, cell proliferation, and remodeling phases [151]. The inflammatory phase occurs in the first few days after injury, during which cells involved in the inflammatory process are activated and play a role in the clearance of cellular debris and secretion of growth factors. The cell proliferation phase is primarily characterized by growth factors that promote the reconstitution of blood vessels and ECM. The remodeling phase, which may last for months or years, is characterized by a decrease in the number of cells and rearrangement of ECM. However, the secretion of some anti-inflammatory factors during this phase may induce fibrosis in the tissues, leading to the formation of adhesions between the tendon and the surrounding tissues and impeding the complete restoration of the tissue's mechanical function. The healing process of damaged tendons is usually long and weak in the early stages due to thin vascularity, lack of cells, and low levels of growth factors [152], [153]. In addition, tendon adhesions that may occur in the later stages often lead to poor healing [154].

Tendon disorders include clinically distinct conditions such as chronic tendinopathy, acute rupture, postoperative repair, and peritendinous adhesion. Progressive tendon-loading exercise is the principal treatment for chronic tendinopathy, whereas nonsteroidal anti-inflammatory drugs and corticosteroid injections mainly provide short-term symptom relief; surgery is reserved for selected ruptures or refractory structural lesions [155]. Because the evidence reviewed here predominantly concerns tendon repair and adhesion prevention, nanoparticle-mediated gene therapy should be positioned as a local perioperative adjunct rather than a replacement for rehabilitation. Temporally controlled regulation of macrophage responses, regenerative signalling, and fibrosis may improve healing quality [156], [157], with outcomes including tendon-specific functional scores, strength, adhesion, imaging integrity, and re-rupture rates.

### 8.2 Gene therapy mechanisms and delivery challenges for tendon diseases

Gene therapy can prolong the effects of treatment and is considered a promising approach for treating tendon injuries. Because the mechanisms of tendon healing are known, the rationale for gene therapy approaches for tendon diseases is to target molecules that play a role in the three phases of the healing process, such as COX-1 and COX-2, which induce inflammatory responses [16]; PDGF, bFGF, and VEGFA, which play a role in vascular regeneration and ECM remodeling [24], [46]; and TGF-β1, which induces fibrosis in the remodeling phase and its downstream mediators Smad3 and PAI-1, among others (Fig. 5) [14], [47], [48].

**Fig. 5:**
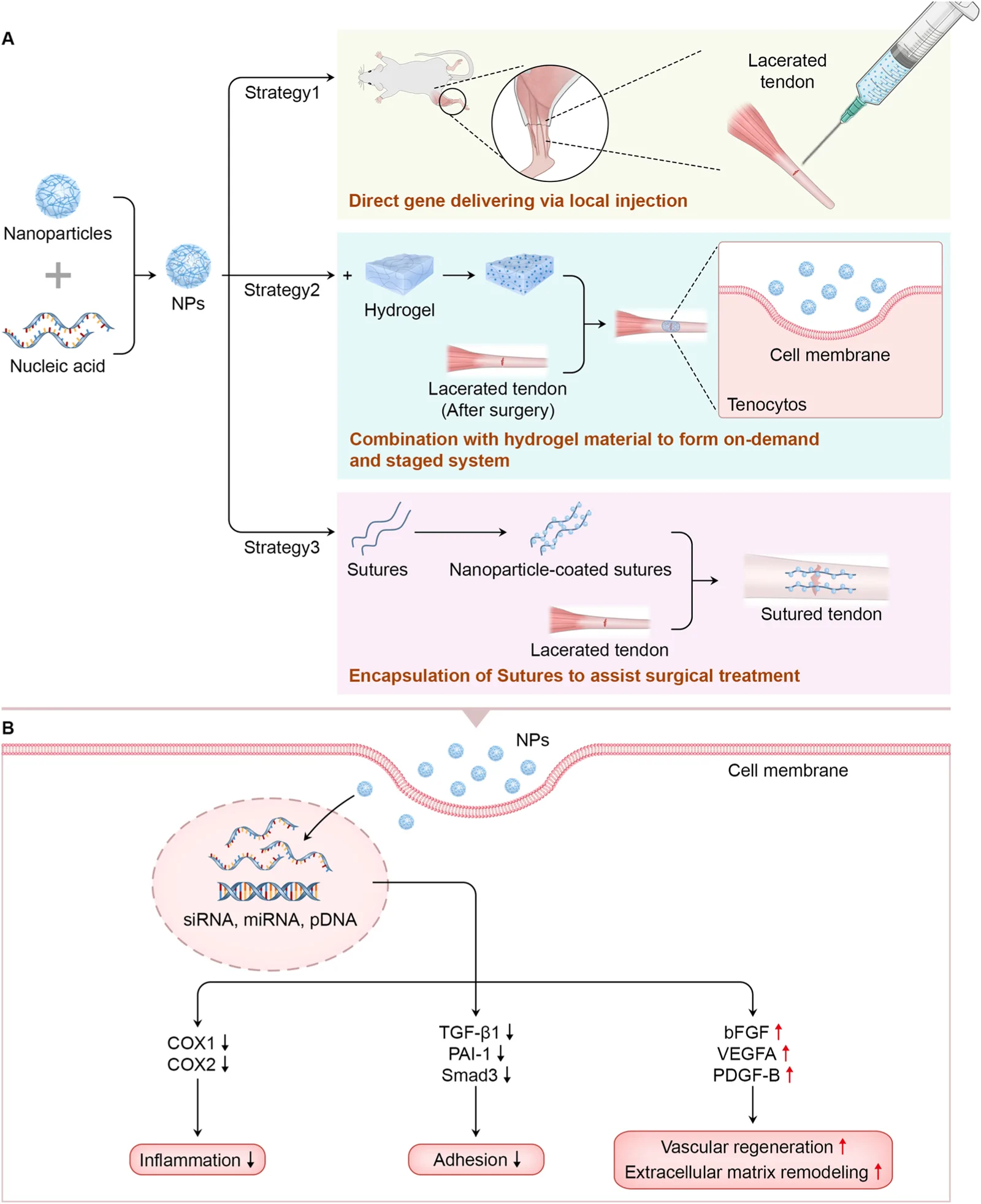
Delivery strategies and implications of nanoparticle delivery system-mediated gene therapy for tendon diseases. (A) Delivery strategies. After nanoparticles are loaded with nucleic acid drugs, strategies for treating tendon diseases include: ① direct injection into damaged tendons, ② combination with hydrogel to form a staged release system, and ③ combination with sutures to assist surgery. (B) The implications on tendon diseases. Nanoparticles enter tendon cells and release nucleic acid drugs such as pDNA, siRNA, and miRNA, which exert anti-inflammation, anti-adhesion, vascular regeneration, and matrix reconstruction through corresponding genes.

However, the efficacy of gene therapy is often limited by the low cellular density and poor vascularization of tendons, as well as the potential for injury associated with conventional injection-based delivery methods [158]. Consequently, gene delivery vectors capable of sustained gene release and high drug-loading capacity are critically important.

### 8.3 Nanoparticle-mediated gene therapy for tendon diseases

The main delivery strategies of nanoparticle-mediated gene therapy for IDD include direct gene delivery via local injection, combination with hydrogel materials to form on-demand and staged-release systems, and encapsulation of sutures to assist surgical treatment (Fig. 5). A series of studies have supported the significant role of nanoparticles in gene transfer in tendon diseases, including gene addition and suppression (Table 5).

**Table 5:** Gene delivery systems for tendon diseases treatment.

Nucleic Acids	Delivery System	In vivo or vitro	Administration method	Ref.
miR-TGF-β1	Polylactic-co-glycolic acid nanospheres	In vivo (chicken model of flexor tendon injury)	Inject locally into the tendon	[14]
miR-TGF-β1	PEI-modified polylactic-co-glycolic acid (PLGA) nanoparticles	In vivo (chicken model of flexor tendon injury)	Inject locally into the tendon	[15]
bFGF and VEGFA pDNA	Polylactic-co-glycolic acid (PLGA) nanoparticles	In vivo (chicken model of flexor tendon injury)	Inject locally into the tendon	[46]
Serpine1 siRNA	Diblock copolymer nanoparticles	In vivo (mouse model of flexor tendon injury)	Inject locally into the tendon	[47]
PDGF-B pDNA	Carboxyl-modified MCM-41 mesoporous silica nanoparticle	In vivo (rat model of flexor tendon injury)	Inject locally into the tendon	[24]
COX-1 or COX-2 miRNA	A novel platform based on the biocompatible polylactic-co-glycolic acid (PLGA) nanoparticle and hyaluronic acid (HA) hydrogel	In vivo (chicken model of flexor tendon injury)	Combination with hydrogel materials	[16]
Smad3-siRNA	Nanoparticles based on guanidinobenzoic acid-modified generation 5 polyamidoamine (G5-GBA) and polycaprolactone (PCL) electrospun nanofibers	In vivo (rat model of flexor tendon injury)	Combination with hydrogel materials	[48]
bFGF and VEGFA pDNA	Suture coated with polylactic-co-glycolic acid (PLGA) nanoparticle	In vivo (chicken and rat model of flexor tendon injury)	Delivery through sutures	[49]

#### 8.3.1 Local injection

Local injection of nanoparticles to deliver genes is an effective approach for treating tendon disorders. For example, Zhou et al. loaded a TGF-β1 miRNA plasmid into PLGA nanoparticles and delivered it to cultured tenocytes and damaged flexor tendons of a chicken model, and found that the delivery vector was able to ensure transgene expression in the tendons for at least 6 weeks without inducing toxicity [14]. Another study by Zhou et al. demonstrated that PLGA nanoparticle/plasmid complexes reduced TGF-β1 expression and tendon adhesion in a chicken model; however, the ultimate strength of the repaired tendon was lower than that of the liposome/plasmid complexes [15]. Yang et al. used PLGA nanoparticles to deliver bFGF and VEGFA genes, which also significantly promoted tendon healing of chicken model [46].

In a study involving mouse tendon cells (mTenocytes) as well as mouse and human mesenchymal stem cells (mMSCs and hMSCs), Malcolm et al. found that the uptake of GAPDH-siRNA and the resultant gene silencing mediated by hydrophobic diblock copolymer nanoparticles were positively correlated with the hydrophobic/hydrophilic ratio of the nanoparticles. Hydrophobic diblock copolymer nanoparticles significantly improved siRNA delivery efficiency and gene silencing with low cytotoxicity to MSCs [159]. Freeberg et al. also developed and adapted a specific design of diblock copolymer nanoparticles for the delivery of Serpine1-siRNA and found that this delivery system was effective in inhibiting Serpine1 expression in tenocytes both in vitro and in vivo, reversing the inhibitory effect of TGF-β1 on MMP activity, and reducing tendon adhesions in mice [47].

Mesoporous silica nanoparticles (MSN) hold great promise for gene therapy of tendon diseases; however, potential toxicity limits their application. Suwalski et al. reported that amino acid- and carboxyl-modified MSNs were capable of delivering the PDGF-B gene in rat Achilles tendon in vivo. Although they were inefficient in transfecting primary tenocytes in vitro, they were able to efficiently transfect and express the gene in the rat Achilles tendon in vivo for at least 2 weeks [24]. Achilles tendon healing was accelerated in the treated group and was significantly better than in the untreated group and the group treated with pPDGF alone. In addition, inflammatory interactions and necrosis were virtually undetectable, implying that the safety of MSN was greatly improved by surface modification. Similarly, histidine-functionalized MSNs designed by Brevet et al. also exhibited higher safety and transfer efficiency [103].

#### 8.3.2 On-demand and staged-release systems

Tendon healing is a lengthy process. Therefore, local delivery systems are required to continuously release their loaded bioactives and over a long period to prolong the therapeutic effect. HA is a natural lubricant that not only exerts its own anti-adhesion and lubrication functions but also achieves slow degradation through the action of various MMPs released during tendon healing. Therefore, combining nanoparticles loaded with gene therapy drugs with HA hydrogels can achieve on-demand and sustained drug release in response to MMPs in the microenvironment of tendon diseases. Zhou et al. synthesized a localized sustained gene delivery system consisting of PLGA nanoparticles and HA hydrogel for the delivery of miRNAs targeting COX-1 or COX-2. This system significantly downregulated the expression of COX-1 and COX-2 in tendon tissues and the surrounding subcutaneous tissues of the chicken model, reduced the formation of tissue adhesions, and improved the sliding function and healing strength of tendons [16]. Cai et al. established a new MMP-2-responsive hydrogel-electrostatic spinning composite barrier consisting of Smad3-siRNA, nanoparticles formed by guanosine benzoic acid-modified 5th generation polyamide (G5-GBA), HA hydrogel, and electrostatically spun nanofibers for on-demand and unidirectional siRNA release. The HA hydrogel incubated with MMPs exhibited on-demand siRNA nanoparticle release behavior and effectively inhibited fibroblast proliferation. Implantation of this composite barrier around tendons in vivo significantly inhibited macrophage polarization and adhesion formation in a rat model [48].

#### 8.3.3 Encapsulation of sutures to assist surgical treatment

Nanoparticle-mediated gene therapy can also be used as an adjunct to surgical treatment and improve tendon healing after surgery. Zhou et al. prepared a nanoparticle/pEGFP-bFGF and pEGFP-VEGFA-encapsulated suture and used it to suture injured tendons. They found that the modified suture significantly promoted tendon healing. This study suggests a genetically modified suture strategy, in which the modified suture can improve tendon healing in chicken and rat models of flexor tendon injury by increasing tendon healing strength, enhancing sliding function, and inhibiting adhesion formation through a mechanism of sustained release of growth factors or therapeutic genes [49].

### 8.4 Effectiveness, shortcomings, and optimization

Earlier tendon nanogene studies primarily used sustained-release systems to suppress fibrosis and reduce adhesion formation [14], [15], [16], [46], [47], [48]. Recent studies have expanded this approach toward modulation of macrophage dysregulation and regeneration of the tendon–bone interface, including Serpine1-siRNA/budesonide lipid–polymer nanoparticles and GAS5-loaded LNPs [6], [104]. These findings suggest a transition from single-target anti-adhesion therapy toward multimodal and compartment-specific treatment. However, differences among tendinopathy, flexor-tendon adhesion, and enthesis-healing models preclude direct comparison, and long-term mechanical safety remains insufficiently characterized.

The core reasons for the effectiveness of the above strategies can be summarized in two points: on the one hand, the carrier characteristics of nanoparticles provide a guarantee for gene delivery. On the other hand, all types of delivery systems can specifically match the physiological needs of tendon healing and reduce the impact on surrounding normal tissues through precise delivery: Local injection can have a rapid effect; the hydrogel sustained-release system can match the long healing cycle of the tendon; and the suture assist system can simultaneously solve the two major problems of surgical suturing and postoperative healing.

Despite significant advances in these treatment strategies, persistent shortcomings across various delivery pathways limit their clinical viability. First, most studies use local injection for nanoparticle-mediated gene delivery; however, due to the density of tendon tissue, the volume of injection reagents is limited, and the injection fluid is prone to leakage, affecting the treatment effect [49]. In the future, it is necessary to build a long-term precision delivery system to achieve precise on-demand release of genes by adjusting the particle size, pore size, and component ratio of nanoparticles, introducing slow-release microsphere structures, or combining with hydrogel materials. Second, the biocompatibility and mechanical properties of materials need to be further improved. For example, unmodified nanoparticles (such as MSNs) are potentially toxic and must be subjected to a series of standardized tests to quantify all potential forms of DNA damage they can cause [24]. In addition, hydrogel materials have poor mechanical properties and are prone to rupture during tendon activity, triggering inflammatory reactions. It is necessary to improve the comprehensive performance of the carrier material and enhance the tensile strength and toughness of the hydrogel through composite nanofibers and other materials so that it can withstand the mechanical load of tendon activity or adjust the asymmetric properties of the hydrogel to match the mechanical needs of different stages of tendon healing [48], [160]. Third, when using nanoparticles to coat surgical sutures, it is necessary to ensure that various materials, including coatings, are non-toxic and at the same time prevent mechanical strength loss or foreign body reactions that may occur after suture modification [49].

## 9 Mechanistic Coupling between successful delivery and disease modulation

### 9.1 Intra-articular retention and tissue-level distribution

Among the disease settings discussed above, OA provides a particularly instructive example of the distinction between administration and tissue-level delivery. Intra-articular administration increases the initial joint concentration of nucleic acids but does not ensure durable exposure of the intended tissue. Free oligonucleotides may be degraded or removed through synovial capillaries and lymphatic drainage. Nanoparticles can prolong residence, yet whole-joint retention, tissue distribution, and persistence of intact cargo are different endpoints. A signal around the knee may arise mainly from the synovium, infrapatellar fat pad, or periarticular tissues rather than cartilage. Whole-joint fluorescence can therefore overestimate delivery to cartilage and can also outlast an intact carrier or cargo [161], [162].

Size, surface charge, matrix affinity, and depot formation determine the balance between residence and penetration. Small carriers generally enter cartilage more readily but may clear faster, whereas larger or hydrogel-associated systems can remain in the joint without reaching deep chondrocytes. Moderate cationic charge promotes interaction with negatively charged glycosaminoglycans, but excessive binding may trap carriers at the surface; disease-related proteoglycan loss further changes this balance. In OA models, MMP13-responsive siRNA micelles increased joint fluorescence exposure and diseased-cartilage localization relative to non-responsive controls [163]. Virus-inspired lipopeptide formulations prolonged siRNA detection to approximately 9 days, and a hyaluronic-acid depot extended the signal beyond 21 days [164]. Accordingly, retention studies should combine longitudinal imaging with tissue-resolved quantification and, ideally, independent labels or sequence-specific assays for the carrier and intact nucleic acid.

### 9.2 Endosomal escape and modality-specific target engagement

After sufficient exposure of the intended tissue has been established, the next question is whether the nucleic acid becomes functional inside the target cells. Cellular uptake is not equivalent to productive intracellular delivery. After endocytosis, the cargo must avoid lysosomal degradation and reach the compartment required for its mechanism. Quantitative imaging of lipid nanoparticle-delivered siRNA has estimated that only about 1–2% of internalized siRNA reaches the cytosol [165].

After sufficient exposure of the intended tissue has been established, successful delivery must be further demonstrated at the intracellular level. Cellular uptake alone does not indicate productive delivery because, following endocytosis, nucleic acid cargo must escape endosomal–lysosomal degradation and reach the subcellular compartment required for its activity. Indeed, quantitative imaging has shown that only approximately 1–2% of siRNA internalized through lipid nanoparticles reaches the cytosol [166]. Endosomal escape should therefore be assessed independently of cellular uptake. Reduced colocalization with EEA1, Rab5, Rab7, LAMP1, or LysoTracker may indicate release from endosomal compartments, although it may also result from cargo degradation or fluorophore dissociation. Complementary approaches, including Galectin-8/9 recruitment, cytosolic fractionation, split-luciferase assays, and single-molecule imaging, can provide more direct evidence of endosomal disruption or cytosolic release [167].

Nevertheless, endosomal escape remains an intermediate delivery step; productive delivery ultimately requires modality-specific target engagement. For siRNA and miRNA, this should be demonstrated by sequence-specific changes in target transcripts and proteins. ASOs require evidence of RNA degradation or splice correction, whereas mRNA and plasmid DNA require expression of the encoded therapeutic protein. CRISPR systems require sequence-resolved confirmation of on-target editing, corresponding changes in target-protein expression, and appropriate off-target analyses. OA studies illustrate how intracellular trafficking and target engagement can be evaluated together. For MMP13-responsive micelles, the siRNA–LysoTracker colocalization coefficient decreased from approximately 0.60 at 1 h to 0.27 at 8 h, followed by suppression of MMP13 transcripts and proteins [168]. Similarly, virus-inspired lipopeptide nanoparticles exhibited low lysosomal colocalization and achieved more than 85% MMP13 silencing in inflammatory chondrocytes [169]. Thus, combining intracellular trafficking measurements with direct molecular evidence of target modulation is necessary to distinguish simple nanoparticle uptake from functionally successful nucleic acid delivery.

### 9.3 Molecular evidence of disease modulation: ECM metabolism, inflammation, and cellular senescence

After sufficient exposure of the intended tissue has been established, the next question is whether the nucleic acid becomes functional inside the target cells. Target engagement becomes disease modulation only when it corrects pathological molecular networks. For cartilage and intervertebral disc degeneration, ECM assessment should include both anabolism, represented by COL2A1/type II collagen, ACAN, and SOX9, and catabolism, represented by MMP13, MMP3, ADAMTS4, and ADAMTS5. COL10A1 or RUNX2 may additionally indicate chondrocyte hypertrophy. Transcript changes should be confirmed at the protein or tissue level because RT-qPCR alone does not establish matrix restoration. In the MMP13-siRNA micelle study, direct MMP13 silencing was accompanied by increased type II collagen and cartilage preservation, thereby extending the delivery evidence presented in Section 9.2 to a downstream ECM response [163].

Inflammatory modulation should pair soluble mediators, such as IL-1β, TNF-α, IL-6, COX-2, and iNOS, with pathway-level readouts. Peptide nanoparticles delivering siRNAs against NF-κB p65 and p100 produced sequence-specific suppression; p65 inhibition reduced chondrocyte apoptosis, proteoglycan loss, and reactive synovitis while altering AMPK, mTORC1, and Wnt/β-catenin signaling [170]. This constitutes stronger mechanistic evidence than an isolated decrease in cytokine concentration.

Senescence also requires a panel rather than a single marker. At least one cell-cycle arrest marker, such as p16 or p21, should be combined with indicators including reduced Ki67/EdU incorporation, SA-β-gal, γH2AX or 53BP1 foci, lamin B1 loss, and senescence-associated secretory phenotype factors. In FAP-positive OA chondrocytes, LNP-mediated FAP siRNA reduced P21 and SA-β-gal together with IL-6, MMP13, NF-κB activation, and cartilage destruction [171]. Current guidelines recommend using at least three complementary markers representing different features of cellular senescence [168]. These therapeutic changes should also be interpreted together with off-target immune responses, including complement activation, interferon signaling, cytokine release, synovial macrophage activation, and immune responses against carrier components such as PEG. Overall, the strongest disease-modulation chain consists of direct target changes followed by coordinated ECM, inflammatory, and senescence responses and, finally, histological or functional improvement.

### 9.4 Bidirectional feedback between disease modulation and nanoparticle fate

The disease-specific chapters above mainly describe how pathological tissue environments influence nanoparticle delivery; however, the relationship may also operate in the opposite direction. Nanoparticle fate is shaped by the disease environment, but effective treatment can subsequently reshape that environment. Proteoglycan loss decreases cartilage fixed-charge density, synovial proteins can modify particle surfaces, and inflammation changes macrophage uptake and vascular or lymphatic clearance. Brown et al. showed that cationic DMAB-PLGA nanoparticles had approximately sixfold greater retention than anionic particles in healthy cartilage, whereas synovial fluid reduced cationic-particle retention by about 50% and OA-like matrix depletion reduced it by as much as 2.9-fold [169]. Thus, performance measured in healthy tissue or buffer may not predict delivery in OA.

The reverse direction is plausible but less frequently tested. ECM restoration could improve the binding of moderately cationic carriers while restricting their deep diffusion. Reduced synovitis could decrease off-target macrophage sequestration but might also weaken macrophage-directed targeting. Responsive systems create an additional negative-feedback possibility: OA-associated ROS or MMP13 can promote hydrogel degradation, ligand exposure, or cargo release, whereas effective siMMP13 treatment reduces the pathological trigger required for subsequent release [163], [166]. This may improve safety but could also result in incomplete dosing. Most studies demonstrate responsiveness before treatment rather than reassessing particle fate after disease correction. Future repeated-dose experiments should therefore compare residence time, cartilage penetration, synovial uptake, cargo release, and systemic drainage before and after a partial therapeutic response. Until such data are available, bidirectional feedback should be presented as a supported mechanistic framework rather than a universally proven phenomenon.

## 10 Translational barriers

Despite the rapid development of nanoparticle-based delivery systems for treating degenerative musculoskeletal diseases, their clinical translation remains limited. This gap cannot be attributed solely to insufficient transfection efficiency but instead reflects a fundamental mismatch between nanoparticle design and the tissue-specific anatomical, physiological, and pathological characteristics of musculoskeletal tissues.

For diseases such as OP, which primarily rely on systemic administration, nanoparticles typically exhibit a pronounced biodistribution bias following intravenous injection. Substantial evidence indicates that most circulating nanoparticles are preferentially sequestered and cleared by the liver and spleen through the reticuloendothelial system, resulting in minimal accumulation in bone tissue [167]. In addition, the structural features of the blood–bone marrow barrier further restrict nanoparticle extravasation into the bone marrow microenvironment, thereby limiting effective interactions with osteogenic target cells and increasing the risk of off-target toxicity [172]. To address these challenges, strategies include: (1) dual/multi-targeting approaches combining bone-affinity molecules (ALN, Asp8) with cell-specific ligands; (2) biomimetic coatings (cell membrane camouflaging) to evade immune clearance; (3) smart responsive carriers activated by bone microenvironment (low pH, specific enzymes); and (4) alternative administration routes (subcutaneous depot systems) enabling sustained systemic delivery [112], [116].

In contrast, OA is commonly treated via intra-articular administration to avoid systemic clearance; however, this approach introduces distinct local barriers to drug absorption. Nanoparticles injected into the joint cavity are rapidly eliminated through synovial fluid turnover and phagocytosis by activated synovial macrophages in the inflammatory microenvironment [173]. At the tissue level, the dense ECM of articular cartilage imposes stringent size and charge constraints on nanoparticles, preventing most gene-loaded carriers from penetrating beyond the superficial cartilage zone [174]. Furthermore, the low metabolic activity of primary chondrocytes and their encapsulation within a pericellular matrix further limit the in vivo effectiveness of non-viral gene transfer [175]. Solution strategies include: (1) size-optimized nanoparticles (<100 nm) for cartilage penetration; (2) cartilage-targeting ligands (CAP, HA, CS) for active targeting; (3) ROS-responsive materials for on-demand release; (4) hydrogel-microsphere platforms for prolonged retention; and (5) tetrahedral framework nucleic acids enabling deep cartilage penetration [12], [140].

IDD represents one of the most challenging microenvironments for nanoparticle-mediated gene delivery. As an avascular tissue, the intervertebral disc relies entirely on passive diffusion for molecular transport, a process that is further compromised by endplate calcification during degeneration. Meanwhile, the acidic pH, elevated oxidative stress, and sustained mechanical loading characteristic of degenerated discs can destabilize nanoparticle structures, promote premature cargo release, or induce mechanical failure, thereby reducing delivery efficiency and the durability of transgene expression [176].

In tendon-related degenerative diseases, dense and highly aligned collagen fibers severely restrict nanoparticle diffusion within the tissue, resulting in markedly lower in vivo transfection efficiency than in vitro observations [177]. More importantly, when osteogenic or pro-regenerative genes such as BMPs are employed, uncontrolled gene expression, carrier leakage, or inflammation-induced stem cell misdifferentiation may lead to heterotopic ossification, representing a major safety concern that limits clinical applicability [178].

Beyond tissue-specific barriers, nanoparticle-mediated gene therapy also faces manufacturing and immunological challenges during clinical translation. To prolong circulation time, nanoparticles are commonly modified with polyethylene glycol (PEG). However, repeated administration of PEGylated carriers can induce the production of anti-PEG antibodies, leading to accelerated blood clearance and reduced therapeutic efficacy [179]. Mitigation strategies include: (1) pre-formed corona approaches using controlled protein pre-coating; (2) zwitterionic surface modification to resist protein adsorption; (3) bio-orthogonal chemistry for active targeting independent of protein corona; and (4) machine learning prediction of protein corona composition enabling rational design [180], [181].

Clinical translation is further constrained by the limited technology readiness of orthopaedic gene therapies and inconsistencies in preclinical evaluation. Many approaches remain confined to small-animal proof-of-concept studies, with insufficient standardization of dose, biodistribution, durability, mechanical outcomes, and long-term safety. Moreover, products combining gene cargoes with nanoparticles, cells, or scaffolds face additional challenges in scalable manufacturing, sterilization, storage, batch consistency, and regulatory evaluation. Progress will therefore require clinically relevant large-animal models, predefined critical quality attributes, and early consideration of manufacturing and regulatory requirements rather than efficacy optimization alone [182], [183], [184], [185].

Overall, these findings indicate that the translational bottleneck of nanoparticle-mediated gene therapy for degenerative musculoskeletal diseases does not arise solely from inadequate delivery efficiency but rather from the complex interplay between delivery systems and tissue-specific biological barriers. Overcoming these challenges require the development of adaptive, environment-responsive, and spatially controllable delivery platforms tailored to distinct musculoskeletal tissues.

## 11 Future perspectives

DMDs significantly affect the quality of life of patients. Although more therapeutic agents are being developed as we delve deeper into their causative mechanisms, reversing tissue damage with existing drugs remains difficult. With the development of gene delivery technology, gene therapy may become an alternative treatment for DMDs in the future. In recent years, nanoparticle-based gene therapy vectors have been expected to be a key strategy to break through the bottleneck of gene delivery by virtue of their potential for low immunogenicity, high payload, and multifunctional modifications. In this review, we first discuss the various types of nanomaterials currently used for gene therapy in DMDs, along with their advantages and disadvantages. We also introduce the delivery strategies and therapeutic effects of nanoparticles in gene therapy for various DMDs, and analyze the core causes of their current effects, existing shortcomings, and future optimization directions.

Currently, gene therapy using nanoparticles as carriers has shown unique potential in the field of degenerative musculoskeletal diseases; however, it also has many limitations. Through a systematic analysis of current research, strengths and weaknesses, coupled with technological development trends, we believe that the following directions are worth in-depth exploration to break through current bottlenecks and expand therapeutic efficacy and applications.

### 11.1 Developing unified gene therapy strategies targeting comorbidities

Degenerative musculoskeletal diseases often do not occur in isolation. For example, knee OA and OP commonly occur together, with intricate connections in epidemiology, pathological mechanisms, and therapeutic strategies [186]. If a single gene therapy regimen could be designed to simultaneously intervene in both diseases, it might significantly improve treatment efficiency and reduce the healthcare burden on patients.

The key to realizing this strategy lies in the precise design of nanoparticles and optimization of nucleic acid delivery systems. When selecting therapeutic targets, attention should be focused on the core signaling pathways shared by both diseases. For instance, the RANKL gene, which regulates osteoclast differentiation, is an ideal candidate; its inhibition can reduce abnormal bone resorption in OP and alleviate subchondral bone destruction, which is common in OA. For carrier construction, the nanoparticle surface can be modified with both chondrocyte-specific peptides and osteoclast-specific antibodies, thereby achieving precise dual-targeting of the two types of pathological cells.

### 11.2 Synergizing biomaterials, cells, and gene therapy

Reliance on a single therapeutic approach is often inadequate. The integration of the physical support of biomaterials, regenerative capacity of cell therapy, and long-lasting regulatory effects of gene therapy holds promise for transformative improvements in the treatment of degenerative musculoskeletal diseases. Preliminary exploration of this integrated strategy has already been conducted. For example, Andersen et al. reported an innovative study in which nanoparticles loaded with specific siRNAs (such as TRIB2 or BCL2L2) were embedded in a biological scaffold, and the scaffold was pre-seeded with MSCs in a maintenance medium for 16 h. When this functionalized scaffold was implanted into mice, it not only provided structural support but also precisely modulated the fate of surrounding mesenchymal stem cells, guiding their differentiation toward osteogenic or adipogenic lineages, ultimately achieving spatially directed tissue regeneration within the restricted region of the implant [26]. This study offers new inspiration for using the spatial design of materials to enable more complex multi-gene modifications or in situ regeneration of multiple cell types. Recent orthopaedic studies further support integrating nanocarriers with cells and instructive scaffolds rather than optimizing each component independently. A recent JOT review showed that nano-embedded growth-factor and stem-cell platforms can improve local retention and regenerative signaling, while also identifying unresolved challenges in dose control, vascularization, mechanical compatibility, and scale-up [187]. For nanogene therapy, scaffolds may therefore function not only as passive depots but also as spatial regulators of cell localization, cargo release, and gene-expression duration.

### 11.3 AI-driven carrier optimization and protein corona prediction

The integration of computational biology and artificial intelligence has significantly accelerated the development of gene therapy carriers [188]. Machine learning models can systematically analyze the complex relationship between the physicochemical properties of nanomaterials and their in vivo delivery efficiency, thereby efficiently screening and optimizing carrier formulations. There have been successful cases in this regard. Using an AI-assisted generative and screening platform, Wang et al. rapidly identified a handful of high-performance new molecules from tens of millions of potential ionizable lipid molecules. One of them even outperformed the commonly used traditional lipid, SM-102, in animal models [189]. Additionally, machine learning models can integrate nanoparticle properties, protein parameters, and in vivo microenvironmental factors to predict the composition of the “protein corona” that forms when nanoparticles enter the bloodstream [180], [181]. This predictive capability is crucial because the “protein corona” can markedly alter the behavior and ultimate therapeutic effect of nanoparticles in vivo. Based on this, we can rationally design specialized carriers tailored to the characteristics of musculoskeletal diseases. Beyond formulation screening, AI could integrate nanoparticle physicochemical properties, protein-corona profiles, and tissue-specific biological responses to identify formulations optimized for bone, cartilage, intervertebral disc, or tendon. However, model interpretability and external validation remain essential, particularly when the training data are not derived from musculoskeletal tissues [190].

### 11.4 Organ-on-a-chip platforms for nanomedicine evaluation

Musculoskeletal organoids and organs-on-chips can provide a disease-relevant intermediate between conventional cell culture and animal models by reproducing mechanical loading, tissue interfaces, perfusion, and inflammatory crosstalk. Recent cartilage- and osteochondral-chip systems have modeled OA-related responses and enabled therapeutic screening under controlled mechanical and vascular conditions [191], [192]. A recent JOT review further emphasized their potential for orthopaedic efficacy, toxicity, and regulatory evaluation, while identifying the need for platform standardization, immune and vascular integration, and cross-laboratory validation [193]. For nanoparticle-mediated gene therapy, these systems could be used to evaluate tissue penetration, cell-specific uptake, endosomal escape, target engagement, and repeated-dose toxicity before large-animal testing.

In conclusion, nanoparticle-mediated gene therapy is reshaping the therapeutic paradigm for DMDs. Despite these challenges, interdisciplinary collaborations to overcome the bottlenecks of targeted delivery and biosafety are expected to drive the development of more effective gene therapies for DMDs and gene nanomedicines from the laboratory to the clinic.

## Authorship statement

All authors have read and agreed to the published version of the manuscript.

Xiaofei Chen: Conceptualization, Investigation, Visualization, Writing – original draft, Writing – review & editing. Jiale Sun: Investigation, Validation, Visualization, Writing – review & editing. Wenqing Xie: Resources, Validation, Writing – review & editing. Djandan Tadum Arthur Vithran: Validation, Visualization. Qidong Zhang: Validation, Writing – review & editing. Jessica M Rosenholm: Resources, Visualization. Yusheng Li: Conceptualization, Funding acquisition, Supervision, Writing – review & editing. Hongbo Zhang: Conceptualization, Funding acquisition, Supervision, Writing – review & editing. Cheng Huang: Conceptualization, Funding acquisition, Supervision, Writing – review & editing.

## Declaration of generative AI in scientific writing statement

During the preparation of this work the authors used ChatGPT and Gemini in order to polish some sentences. After using this tool, the authors reviewed and edited the content as needed and take full responsibility for the content of the publication.

## Funding

This work was supported by the 10.13039/100014717National Natural Science Foundation of China (82372500, 82472522, and 92268115); the 10.13039/501100004826Beijing Natural Science Foundation Haidian Original Innovation Joint Fund (L252152); the 10.13039/501100012166National Key R&D Program of China (2023YFC3603400); the Projects of International Cooperation and Exchanges of the 10.13039/100017053National Natural Science Foundation of China (W2421123); the 10.13039/501100002767Hunan Provincial Science Fund for Distinguished Young Scholars (2024JJ2089); and the Independent Exploration and Innovation Project for Postgraduate Students of Central South University (2024ZZTS0163).

## Declaration of competing interest

The authors declare that they have no known competing financial interests or personal relationships that could have appeared to influence the work reported in this paper.
